# Stem Cell Transplantation in Traumatic Spinal Cord Injury: A Systematic Review and Meta-Analysis of Animal Studies

**DOI:** 10.1371/journal.pbio.1001738

**Published:** 2013-12-17

**Authors:** Ana Antonic, Emily S. Sena, Jennifer S. Lees, Taryn E. Wills, Peta Skeers, Peter E. Batchelor, Malcolm R. Macleod, David W. Howells

**Affiliations:** 1 Department of Medicine, University of Melbourne Lance Townsend Building, Austin Hospital, Heidelberg, Victoria, Australia; 2 Florey Institute of Neuroscience and Mental Health, Victoria, Australia; 3 Division of Clinical Neurosciences, University of Edinburgh, Western General Hospital, Edinburgh, United Kingdom; Oxford University, United Kingdom

## Abstract

Spinal cord injury (SCI) is a devastating condition that causes substantial morbidity and mortality and for which no treatments are available. Stem cells offer some promise in the restoration of neurological function. We used systematic review, meta-analysis, and meta-regression to study the impact of stem cell biology and experimental design on motor and sensory outcomes following stem cell treatments in animal models of SCI. One hundred and fifty-six publications using 45 different stem cell preparations met our prespecified inclusion criteria. Only one publication used autologous stem cells. Overall, allogeneic stem cell treatment appears to improve both motor (effect size, 27.2%; 95% Confidence Interval [CI], 25.0%–29.4%; 312 comparisons in 5,628 animals) and sensory (effect size, 26.3%; 95% CI, 7.9%–44.7%; 23 comparisons in 473 animals) outcome. For sensory outcome, most heterogeneity between experiments was accounted for by facets of stem cell biology. Differentiation before implantation and intravenous route of delivery favoured better outcome. Stem cell implantation did not appear to improve sensory outcome in female animals and appeared to be enhanced by isoflurane anaesthesia. Biological plausibility was supported by the presence of a dose–response relationship. For motor outcome, facets of stem cell biology had little detectable effect. Instead most heterogeneity could be explained by the experimental modelling and the outcome measure used. The location of injury, method of injury induction, and presence of immunosuppression all had an impact. Reporting of measures to reduce bias was higher than has been seen in other neuroscience domains but were still suboptimal. Motor outcomes studies that did not report the blinded assessment of outcome gave inflated estimates of efficacy. Extensive recent preclinical literature suggests that stem-cell–based therapies may offer promise, however the impact of compromised internal validity and publication bias mean that efficacy is likely to be somewhat lower than reported here.

## Abstract 2

A systematic analysis of the literature shows that stem cell implantation can improve function in animal models of spinal cord injury, depending on the methods used.

## Author Summary

Spinal cord injury is an important cause of disability in young adults, and stem cells have been proposed as a possible treatment. Here we systematically assess the evidence in the scientific literature for the effectiveness of stem-cell–based therapies in animal models of spinal cord injury. More studies reported effects on the ability to move (“motor outcomes”) than on sensation (“sensory outcomes”). Overall, treatment improves both sensory and motor outcomes, and for sensory outcome there was a dose–response effect (which suggests an underlying biological basis). Although more measures were taken to reduce the risk of bias than in other areas of translational neuroscience, unblinded studies tended to overstate the effectiveness of the treatment. The variability observed between the studies is not explained by differences in the stem cells used, but does seem to depend on the different injury models used to emulate human spinal cord injury. This suggests that the mechanism of injury should be an important consideration in the design of future clinical trials. Furthermore, open questions arise about the use of immunosuppressive drugs, and efficacy in female animals; these should be addressed before proceeding to clinical trial.

## Introduction

Stem cells, from which all tissues can be generated, offer the potential to reconstitute tissues damaged by injury and disease. However, realising this potential will demand a detailed knowledge of the genetic and internal environmental cues that specify a cell's type, location, and interaction with its neighbours. It will also require a thorough understanding of stem cell behaviour in the context of lesioned or damaged tissues.

Stem cell transplantation was pioneered in the 1950s using haematopoietic stem cells to repopulate the bone marrow in patients with cancers of the blood and bone marrow [1]. Such is the success of this approach that an estimated 50,000 of these transplants are performed each year [2]. As understanding of stem cell biology has increased, so too has the ambition for restoring more complex tissues. In animal models, hepatocytes derived from stem cells can be engrafted into the damaged liver [3], and lineage-specific stem cells can repair damaged cornea [4],[5]. Recent studies also demonstrate the generation of artificial tissues with key features of complex solid organs including blood vessels [6], heart [7]–[9], lung [10], and kidney [11]. Even in the CNS, where the breadth of cell types and the complexity of their interactions are maximal, stem cell implants appear able to integrate into the existing circuitry [12]–[14]. In patients, lineage-specific stem cells have been reported to show efficacy in the regeneration of craniofacial bones [15] and of damaged cornea [5].

Integration into the host environment and tissue reconstruction are not the only potentially relevant biological effects of stem cells. Immunomodulatory effects of stem cells appear to reduce rejection of kidney transplants [16],[17], corneal allografts [18], and composite tissue hemi-facial allografts [19]. In the CNS, stem cells are reported to provide immunomodulatory and neuroprotective effects in models of diseases as disparate as retinopathy [20], neuronal ceroid lipofuscinosis [21], motor neuron disease [22],[23], Parkinson's disease [24], multiple sclerosis [25],[26], stroke [27]–[29], and spinal cord injury [30],[31].

There is now considerable preclinical literature on the possible benefits of stem-cell–based therapies following traumatic spinal cord injury. Stem cells may assist recovery through limitation of secondary injury, re-myelination, formation of new neuronal connections, and alteration of the inhibitory environment. However, it is unclear which type of cells and from what source are best to implant, how many are needed, whether immunosuppression should be used, and whether the implanted cells need to be modified to enhance particular desirable characteristics. It is also unclear whether the magnitude of integrative and protective effects is large enough to be potentially clinically meaningful. We also do not know whether reports of efficacy in animal models are potentially biased in favour of positive results.

Here, we report a systematic review, meta-analysis, and meta-regression of data from controlled *in vivo* studies testing the efficacy of stem cells as a treatment in animal models of spinal cord injury. Our objectives are (i) to establish a summary estimate of the efficacy of stem cells in animal models of traumatic spinal cord injury, (ii) to ascertain the conditions under which animal experiments demonstrate greatest efficacy, and (iii) to determine any effect of study quality on reported efficacy.

## Results

### Study Characteristics

Electronic searching identified 156 full publications that met our prespecified inclusion criteria (Table S1). Forty-five different stem cell types had been investigated, from which over a third were derived from adult rats. The duration of experiments following the induction of SCI ranged from 7 d to 6 mo.

One publication [32] with two individual comparisons involving 36 animals reported the effect of autologous bone marrow stromal cells on motor score. We included this publication in the overall assessment of the prevalence of the reporting of measures taken by the original authors to reduce the risk of bias in their experiments. However, because this was the only paper to report the effects of autologous (rather than allogeneic) stem cells, we did not analyse this further, focussing instead on allogeneic stem cells.

**Figure 1:**
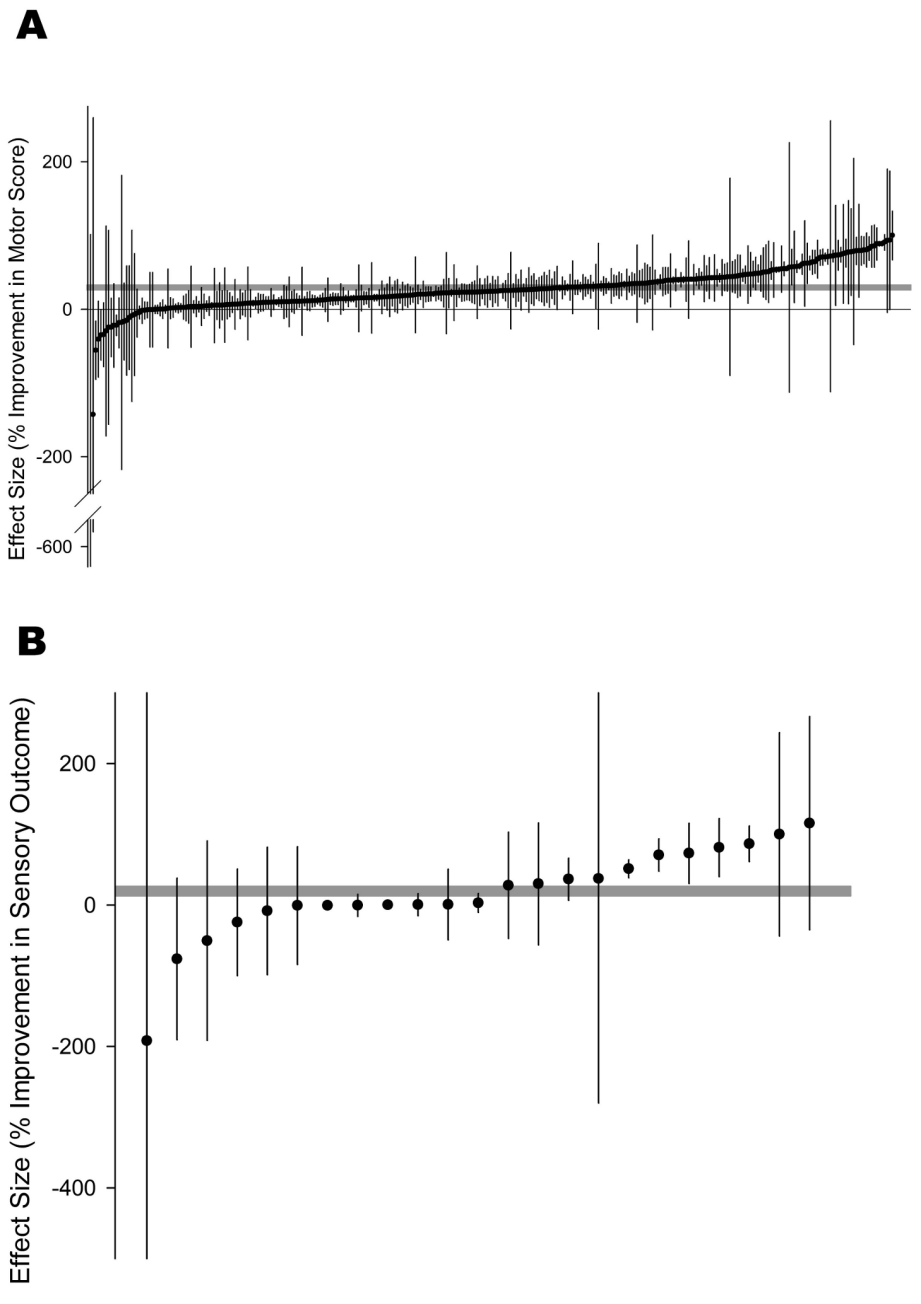
Summary of data included in meta-analysis of use of stem cells to treat spinal cord injury with individual comparisons ranked according to their effect on (A) % improvement in motor score and (B) % improvement in sensory score. The shaded grey bar represents the 95% confidence limits of the global estimate. The vertical error bars represent the 95% confidence intervals for the individual estimates.

One hundred and fifty-five publications reported the effect of allogeneic stem cells in 317 individual comparisons; 380 different motor outcomes were reported and because more than one motor outcome was reported for some individual comparisons we nested (see Methods) these into 312 individual comparisons involving 5,628 animals (Figure 1A). Six different tests were used to assess motor score: the Basso, Beattie and Bresnehan locomotor rating scale (BBB; [33]), the Basso mouse scale (BMS; [34]), the Tarlov scale [35], the forelimb placing test [36], the staircase test [37], and the mouse hind limb motor score [38]. Sixty-one sensory outcomes were reported; we excluded six outcomes that tested sensation in unaffected limbs. In 10 outcomes that used the same test at different intensities in the same cohort of animals, we only included the median intensity. Therefore, we report data on sensory outcome reported in 45 experiments nested into 24 comparisons using 473 animals (Figure 1B). In 18 cohorts both motor and sensory outcomes were reported.

### Risk of Bias

We describe the reporting of study quality checklist items reported for each included publication in Table S2. All studies included in this analysis came from peer-reviewed papers; while we identified a number of potentially relevant abstracts, none of these reported data in sufficient detail to be included. One hundred and eleven of 156 publications (71%) reported compliance with animal welfare regulations, and 25 (16%) reported whether or not a conflict of interest existed.

Allocation concealment was reported in 14 of 156 publications (9%). Random allocation to treatment group (72, 46%) and blinded assessment of outcome (72, 46%) were reported more frequently in these publications than in the modelling of other neurological disorders [39]–[42], but the reporting of a sample size calculation (less than 1%) was consistent with the proportions observed elsewhere (Table 1). No publication reported all four of these measures to minimise bias.

**Table 1:** Reporting of study quality criteria.

Item	SCI	FCI [41]	EAE [42]	PD [40]	AD [39]
Random allocation to group	46%	36%	9%	16%	15%
Blinded assessment of outcome	46%	29%	16%	15%	21%
Sample size calculation	1%	3%	<1%	<1%	0%
Compliance with animal welfare regulations	71%	57%	32%	40%	54%
Statement of a potential conflict of interest	16%	23%	6%	2%	11%

Abbreviations: AD, Alzheimer's disease; EAE, experimental autoimmune encephalomyelitis; FCI, focal cerebral ischaemia; PD, Parkinson's disease; SCI, spinal cord injury.

Despite the reported benefits of hypothermia in SCI [43]–[45], in other animal models of neurological disease [46] and in humans with ischaemic neurological injury [47],[48], only 33 (21%) studies described controlling temperature during the experiments.

**Figure 2:**
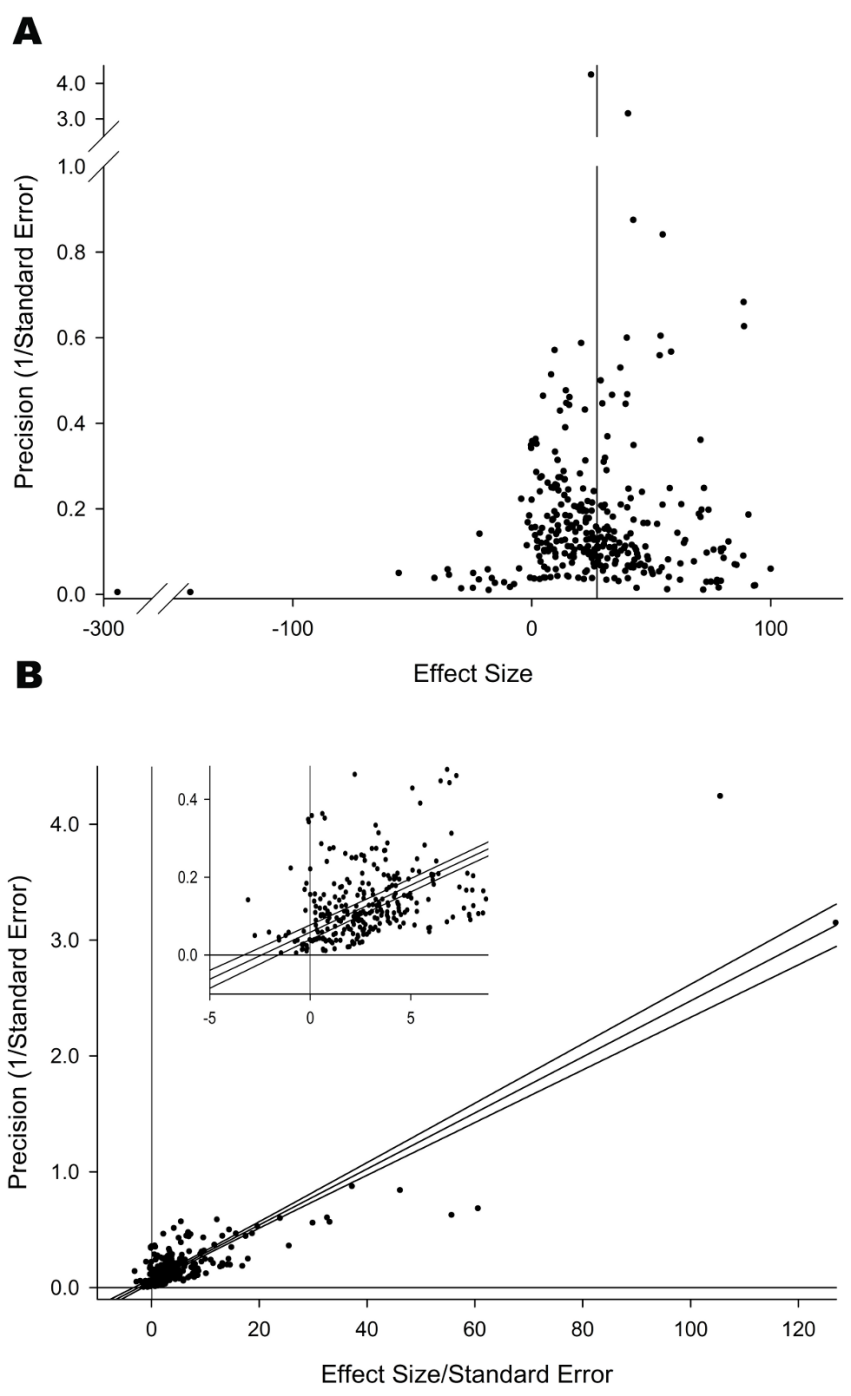
Assessment of publication bias shown with (A) Funnel plot and (B) Egger regression.

There were only sufficient data to assess publication bias in studies using allogeneic stem cells where outcome was measured as a motor score. Small study bias was suggested with asymmetry of the funnel plot (Figure 2A) and Egger regression (Figure 2B) but not by Trim and Fill.

### Meta-Analysis

As expected, our search identified a diverse range of experiments. There was substantial between-study heterogeneity for studies using allogeneic stem cells both where outcome was measured as a motor score [heterogeneity (χ^2^) = 9,735, 311 degrees of freedom (df), *p*<10^−99^; effect size, 27.2% improvement in outcome [95% confidence interval, 25.0%–29.4%]; 312 comparisons) and as a sensory outcome (χ^2^ = 183, *df* = 23, *p*<10^−26^; effect size, 26.3% [7.9%–44.7%]; 24 comparisons).

#### Motor score in experiments using allogeneic stem cells

In meta-regression, eight study characteristics accounted for a significant proportion of the between-study heterogeneity in studies reporting a change in motor score (Table 2). More influence was apparent for factors related to the lesion model than those related to stem cell biology. There was no detectable effect of stem cell dose, derivation (adult or embryonic), manipulation in culture (genetic, growth factor, antibiotic), number of passages in culture, method of stem cell selection prior to implantation, route of administration, frequency of administration, the presence or absence of a supporting scaffold, time of assessment, anaesthetic used, or temperature regulation during surgery.

**Table 2:** Study characteristics accounting for heterogeneity of motor score.

Motor Score		Effect size % (95% CI)	Number of Animals	Number of Comparisons	Adjusted R^2^	*p*<
Pooled estimate		27.2 (25.0–29.4)	5,628	312		
NBS	Motor tests	−8.1 (−37.7–21.4)	49	3	12.24%	0.00001
	Staircase	−2.0 (−43.3–39.3)	12	1		
	BMS	24.5 (11.2–37.7)	196	10		
	Multiple tests	24.5 (17.8–31.2)	1,053	56		
	BBB	26.7 (23.9–29.4)	4,042	228		
	Forelimb placing test	47.9 (18.8–77.1)	76	5		
	Tarlov	73.1 (57.5–88.7)	200	9		
Location of injury	Cervical	32.2 (12.2–52.3)	156	13	10.64%	0.00001
	Lowerthoracic/lumbar	48.1 (39.7–56.5)	456	28		
	Midthoracic	24.9 (22.3–27.5)	5,016	271		
Sex	Female	22.9 (19.6–26.3)	2,906	171	9.69%	0.00001
	Male	27.4 (21.7–33.1)	1,704	87		
	Unknown	35.7 (27.9–43.5)	676	37		
	Both	48.7 (37.6–59.7)	341	17		
Immunosuppression	Cyclosporine A/MP	−11.5 (−92.5–69.5)	12	1	5.83%	0.0026
	FK506	11.6 (−8.0–31.2)	80	6		
	Cyclosporine A	19.6 (13.7–25.4)	1,242	78		
	None	30.2 (27.2–33.1)	4,259	226		
	Cyclophosphamide	44.4 (−0.8–89.7)	36	1		
Method used to induce SCI	Impactor with spacer	11.6 (−11.3–34.5)	79	5	4.40%	0.0115
	Aneurysm clip	18.7 (7.9–29.4)	356	20		
	Impactor	24.1 (20.4–27.8)	2,768	144		
	Unknown	27.8 (19.7–36)	665	35		
	Balloon compression	28.4 (15.8–41.1)	235	14		
	Compression weight	30.1 (20.3–39.9)	544	25		
	Blade	33.5 (26.4–40.6)	682	52		
	Scissors	42.5 (30–54.9)	278	16		
	Filament	79.2 (4.8–53.6)	20	1		
Source of cells	Cell line	41.1 (25.1–57.1)	131	7	4.34%	0.0034
	Human	28.0 (21.6–34.3)	1,483	77		
	Mouse	18.0 (11.2–24.8)	877	56		
	Rat	29.2 (25.9–32.6)	3,136	172		
Type of injury	Contusion	23.8 (20.1–27.5)	2,847	149	3.44%	0.0073
	Compression	25.8 (18.8–32.8)	1,135	59		
	Transection	30.5 (24.1–37))	928	65		
	Hemisection	37.7 (29.1–46.2)	717	38		
Blinded assessment of outcome	Not blinded	30.3 (26.8–33.8)	2,975	165	2.21%	0.01
	Blinded	23.6 (18.5–28.7)	2,653	147		

**Figure 3:**
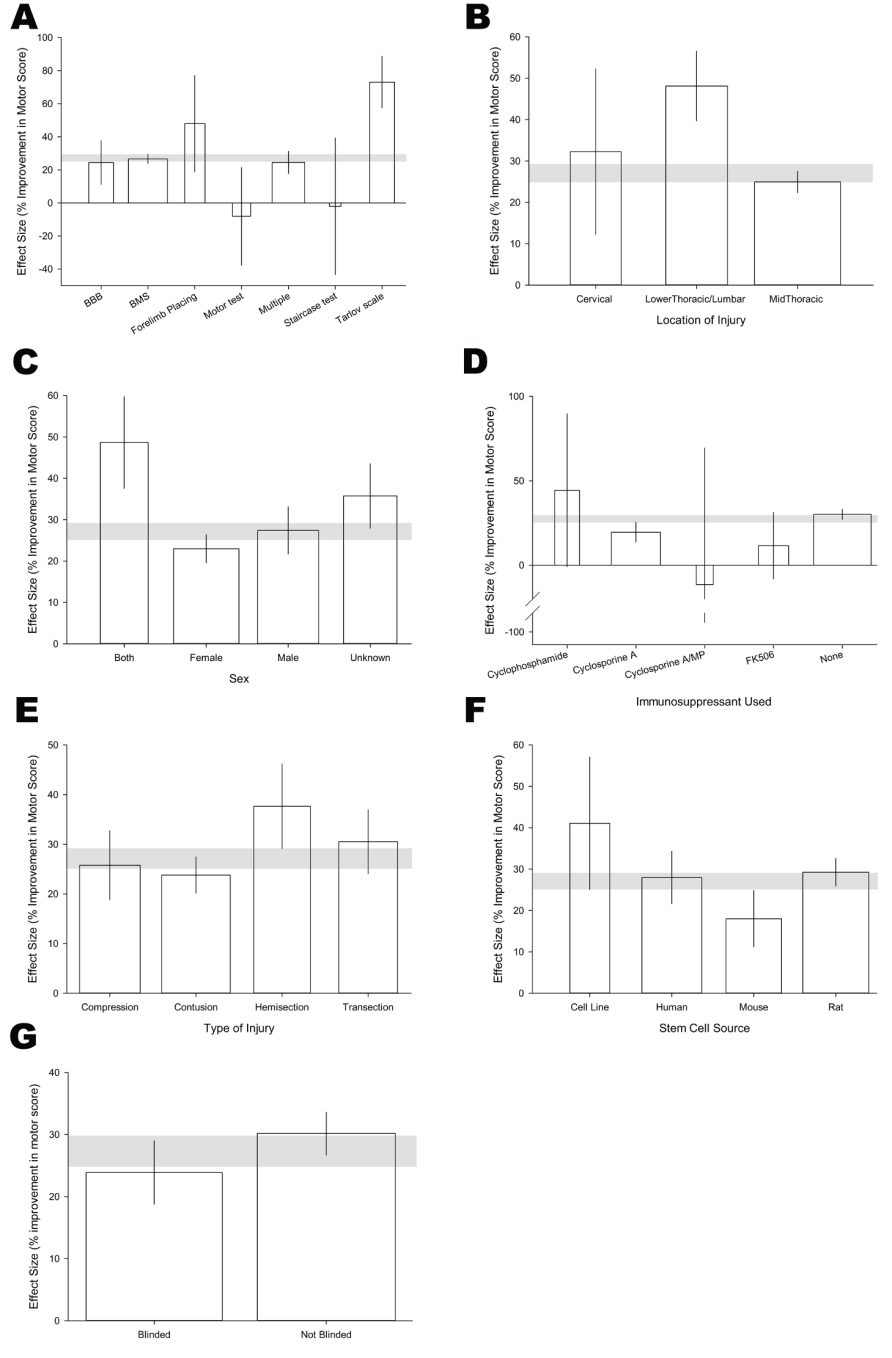
Study characteristics which account for heterogeneity of total motor dataset. (A) Behavioural test used, (B) location of injury, (C) sex of animals, (D) immunosupressant used, (E) type of Injury, (F) stem cell source, and (G) effect of blinding. The shaded grey bar represents the 95% confidence limits of the global estimate. The vertical error bars represent the 95% confidence intervals for the individual estimates.

The neurobehavioural test used (Figure 3A) accounted for most of the observed heterogeneity (adjusted R^2^ = 12.2%, *p*<0.00001). Seventy percent of the data (228 comparisons, 4,042 animals) was obtained using the BBB locomotor rating scale and suggested an improvement in outcome of 26.7% (95% CI, 23.9–29.4). Other tests contributed at most 3.5% of the data; the BMS (10 comparisons, 196 animals) gave results similar to those observed using the BBB scale (24.5%, 11.2–37.7), while the Tarlov (9 comparisons, 200 animals) and forelimb placing tests (5 comparisons, 76 animals) suggested larger effects (73.1%, 57.5–88.7 and 47.9%, 18.8–77.1, respectively). The staircase (1 comparison, 12 animals) and mouse hind limb motor score (3 comparisons, 49 animals) tests reported no significant overall effects. Where multiple tests were used (in 20% of animals) the detected effect size was not different to when BBB or BMS were used alone.

Location of injury (Figure 3B) accounted for 10.6% (adjusted R^2^, *p*<0.00001) of the observed heterogeneity, with larger improvements detected with the most caudal (low thoracic and lumbar) spinal cord lesions compared with other locations.

Sex accounted for 9.7% (adjusted R^2^, *p*<0.00001) of observed heterogeneity, with efficacy higher in males (27.4%, 21.7–33.1, 1,704 animals) compared with females (22.9%, 19.6–26.3, 2,906 animals). Where sex was not reported and where both sexes were used (together 18% of the data), substantially higher estimates of effect size were observed (Figure 3C).

Efficacy was lower when immunosuppression was used (adjusted R^2^ = 5.8%, *p*<0.005). For cyclosporine A [78 comparisons, 1,242 (22% of total) animals], efficacy was 19.6% (13.7–25.4) compared with 30.2% (27.2–33.1) in 226 comparisons and 4,259 animals where no immunosuppression was used. Efficacy also appeared smaller in a small number of experiments [6 comparisons, 80 (1.4%) animals] using FK506 (Figure 3D).

The approach used to induce injury had a smaller but significant effect (adjusted R^2^ = 3.4%, *p*<0.01, Figure 3E). The most common approach was contusion injuries [149 comparisons, 2,847 animals; 23.8% improvement, (20.1–27.5)] with compressive approaches providing improvements of a similar magnitude [59 comparisons, 1,135 animals; 25.8% (18.8–32.8)]. Slightly higher estimates of effect size were obtained when the cord had been transacted [65 comparisons, 928 animals; 30.5% (24.1–37.0)] or hemisected [38 comparisons, 717 animals; 37.6% (29.1–46.2)].

Efficacy was highest with treatment strategies using cell lines (7 comparisons, 131 animals) rather than primary cells, and amongst primary cells those derived from mice were the least effective (Figure 3F, adjusted R^2^ = 4.3%, *p*<0.005).

Efficacy was lower in studies reporting the blinded assessment of outcome [147 comparisons, 2,653 animals, 23.6% (18.5–28.7)] than in those that did not [165 experiments, 2,975 animals, 30.3% (26.9–33.8); Figure 3G; adjusted R^2^ = 2.2%, *p*<0.01]. No effect was seen for reporting of allocation concealment, randomisation, or sample size calculations.

#### Motor score subanalyses

A large proportion of the data (115 comparisons, 2,165 animals) were obtained from rats implanted with allogeneic stem cells, after injury created with an impactor, at the midthoracic level and assessed by the BBB test, where the sex of the animal was explicitly stated. This large and experimentally homogeneous subset of the data was analysed separately to establish whether a clearer picture of the key determinants of stem cell biology and implantation emerged.

Heterogeneity was reduced from 9,735 (χ^2^) over 312 individual comparisons to 1,420 over 115 comparisons, confirming the validity of this approach. As in the full analysis, stem cell dose, number of passages during culture, the presence of additional antibiotics or growth factors in the culture medium, selection methodology, the use of adult or embryonic stem cells and the species of origin, route of administration, presence of a supporting scaffold, and prior differentiation or transfection of the stem cells had no significant effect.

**Figure 4:**
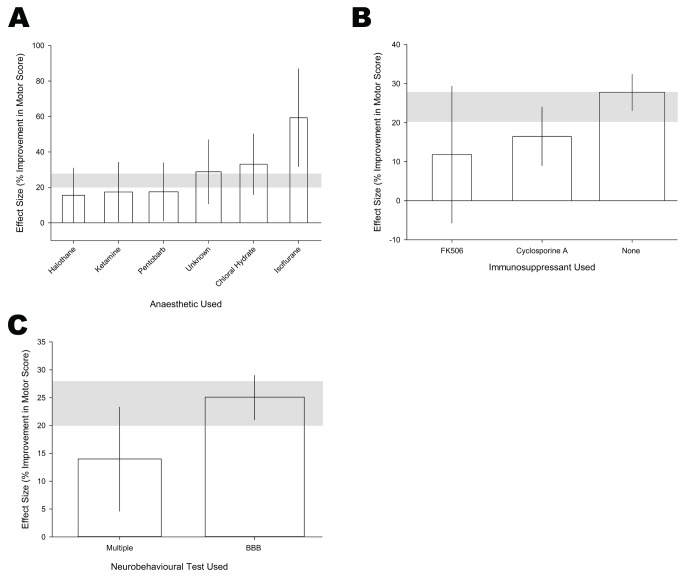
Study characteristics that account for heterogeneity of motor data subanalysis when only data from rats implanted with allogeneic stem cells after injury created with an impactor at the midthoracic level and assessed by BBB. (A) Anaesthetic used, (B) immunosupressant used, and (C) influence of additional behavioural testing on BBB. The shaded grey bar represents the 95% confidence limits of the global estimate. The vertical error bars represent the 95% confidence intervals for the individual estimates.

In this subpopulation of comparisons (Table 3) the anaesthetic used accounted for a high proportion of the heterogeneity (adjusted R^2^ = 16.3%, *p*<0.001). Isoflurane was infrequently used (3 comparisons, 47 animals) and was associated with the largest improvement in outcome. Of the most commonly used anaesthetics, chloral hydrate [21 comparisons, 417 animals, 33.0% (16.0–50.1)] was associated with the largest effect size (Figure 4A).

**Table 3:** Study characteristics accounting for heterogeneity of motor score subanalysis.

Motor Score		Effect Size % (95% CI)	Number of Animals	Number of Comparisons	Adjusted R^2^	*p*<
Pooled estimate		24.1 (20.1–28.1)	2,165	115		
Anaesthetic	Halothane	15.5 (0.3–30.8)	147	6	16.3%	0.0007
	Ketamine	17.4 (0.6–34.2)	508	31		
	Pentobarb	17.5 (1.1–33.9)	740	39		
	Unknown	28.8 (10.8–46.8)	265	13		
	Chloral Hydrate	33.0 (16.0–50.1)	417	21		
	Isoflurane	59.2 (31.8–86.7)	47	3		
Time of assessment	−1.7(−2.8 to −0.6) for each 1 week delay in assessment				11.0%	0.002
Immunosuppression	FK506	11.8 (−6.0–29.5)	80	6	10.42%	0.0064
	Cyclosporine A	16.5 (9.0–24.0)	675	40		
	None	27.8 (23.1–32.4)	1,410	69		
NBS	Multiple	14.0 (4.7–23.3)	473	22	5.0%	0.02
	BBB	25.1 (21.0–29.1)	1,692	93		

The interval from lesioning to outcome assessment accounted for 11.0% of the heterogeneity such that absolute effect size fell by 1.7% for every additional week of delay to outcome assessment. The presence of immunosuppression also accounted for a large proportion of the heterogeneity in this constrained dataset (adjusted R^2^ = 10.4%, *p*<0.01); both cyclosporine A and FK506 substantially reduced the benefit derived from stem cells (Figure 4B). BBB scores were lower in experiments where other tests had also been reported [22 comparisons, 473 animals, 14.0% (4.7–23.3)] than where BBB was reported alone [93 comparisons, 1,692 animals, 25.1% (21.0–29.1); Figure 4C, adjusted R^2^ = 5.0%, *p*<0.02]. There was no impact of whether stem cells were given once, at multiple times, or by continuous infusion; the sex of the animals; or the reporting of randomisation, allocation concealment, or blinded assessment of outcome.

A second subanalysis of the motor dataset was performed to examine whether restriction of the analysis to higher quality studies appreciably altered the results. This analysis was hampered by the paucity of truly high-quality data. None of the contributing papers reported each of four key measures of internal validity (randomisation, blinded assessment of outcome, allocation concealment, and sample size calculation), and only 20 individual comparisons came from papers describing three of the four. As a compromise we analysed the 25% of the motor dataset that reported having both randomisation and blinding.

Restricting the analysis in this way reduced the number of animals assessed from 5,628 to 1,466 and heterogeneity fell from 9,735 to 945 (χ^2^). Despite this, the key features of both the full and the subanalysis are the same. The characteristics of the animal model still have more impact than the type of cells implanted (Tables 2 and 4).

**Table 4:** Study characteristics accounting for heterogeneity of motor score—Randomised and blinded subset.

Motor Score		Effect Size % (95% CI)	Number of Animals	Number of Comparisons	Adjusted R^2^	*p*<
Pooled estimate		24.7(20.2–29.3)	1,466	79		
Method used to induce SCI	Aneurysm clip	−1.7 (−44.3–40.9)	18	2	41.2%	0.0000001
	Balloon compression	19.8 (0.1–39.4)	79	6		
	Compression, weight	21.9 (8–35.8)	253	8		
	iridesctomy scissors	57.6 (40.2–75)	115	5		
	Impactor	14.6 (7.9–21.3)	680	34		
	Knife	42 (29.2–54.8)	169	11		
	Unknown	23.1 (11.2–35)	152	13		
Type of injury	Compression	20.2 (7.2–33.2)	350	16	25.46%	0.0007
	Contusion	14.7 (7.4–21.9)	680	34		
	Hemisetion	41.1 (27.354.9)	240	12		
	Transection	32.9 (21.1–44.8)	196	17		
Location of injury	LowerThoracic/Lumbar	53.8 (37.9–69.7)	78	7	23.68%	0.000001
	Midthoracic	21.3 (16.4–26.2)	1,388	72		
NBS	BBB	24.2 (19.1–29.3)	1,165	66	22.51%	0.0002
	Multiple tests	15.2 (2.1–28.4)	241	11		
	Tarlov	84.5 (54.7–114.3)	60	2		
Dose		7.08 (3.52–1.06) for each additional million cells			21.85%	0.000001
Sex	Both	53.8 (37.2–70.4)	78	7	21.28%	0.0015
	Female	21 (14.6–27.4)	813	43		
	Male	22.6 (12.3–32.8)	553	28		
	Unknown	3.2 (−39.2–45.6)	22	1		
Allocation concealment	Concealed	37.1 (26.3–47.9)	368	20	2.21%	0.01
	Not concealed	19.4 (13.7–25.1)	1,098	59		
Cell culture medium	Antibiotic+Growith Factor	23.2 (7.4–39.1)	240	13	10.84%	0.031
	Growth Factor	19.2 (12–26.3)	663	36		
	Other	26 (14.4–37.7)	479	22		
	Unknown	44.1 (27.7–60.6)	84	8		
Cell manipulations	Differentiation	10.4 (−3.1–23.9)	33	17	10.26%	0.0224
	Diff.+Transfection	19.7 (−8.8–48.3)	33	2		
	None	27.3 (19.1–35.6)	646	28		
	Transfection	22.8 (8.9–36.7)	272	18		
	Unknown	36 (22.4–49.6)	193	14		

Immunosuppression no longer has an effect on heterogeneity and the effect size in animals immunosuppressed with cyclosporine-A [mean, 24.3; 95% CI, 13.2–35.3] is the same as in animals where immune suppression is not used (mean, 24.9; 95% CI, 18.3–31.6). Allocation concealment emerges as significant, though not in the expected direction. Also the type of cell culture medium and type of cell manipulation prior to implantation also begin to have an impact, but it should be noted that in both cases it is the experiments where the precise conditions are “unknown” that report the greatest effect. In the subanalysis, the mean number of cells implanted is substantially lower than in the full analysis (6.3×10^5^ versus 7.4×10^8^), and a dose–response relationship is evident.

#### Sensory score in experiments using allogeneic stem cells

While motor behaviour was relatively unaffected by most factors specific to stem cell biology, the reverse was true for studies reporting a change in sensory outcome (Table 5).

**Table 5:** Study characteristics accounting for heterogeneity of sensory score.

Sensory Outcome		Effect Size % (95% CI)	Number of Animals	Number of Comparisons	Adjusted R^2^	*p*<
Pooled estimate		26.3 (7.9–44.7)	473	23		
Cell manipulation	Differentiation	79.9 (34.8–125)	156	3	61.27%	0.0049
	Transfection	12.1 (−23.1–47.3)	131	8		
	None	42.5 (12.7–72.3)	156	10		
	Unknown	0.0 (−43.5–43.5)	30	2		
Anaesthetic	Isoflurane	81.0 (37.0–125.1)	161	3		
	Ketamine	12.9 (−11.3–37.1)	131	11		
	Pentobarbital	17.5 (−16.7–51.8)	104	5	42.79%	0.048
	Halothane	−15.3 (−318.1–287.5)	24	2		
	Unknown	17.7 (−53.7–89)	53	2		
Dose	27.8 (5.6–50.0) for each increment of 1×10^6^cells				31.72%	0.017
Sex	Male	39.7 (3.9–75.4)	347	11	21.48%	0.03
	Female	−0.3 (−29.5–28.9)	126	12		
Route of delivery	Intraspinal cord	20.4 (2.5–38.4)	428	21	19.25%	0.046
	Intravenous	77.2 (21.6–132.8)	45	2		

**Figure 5:**
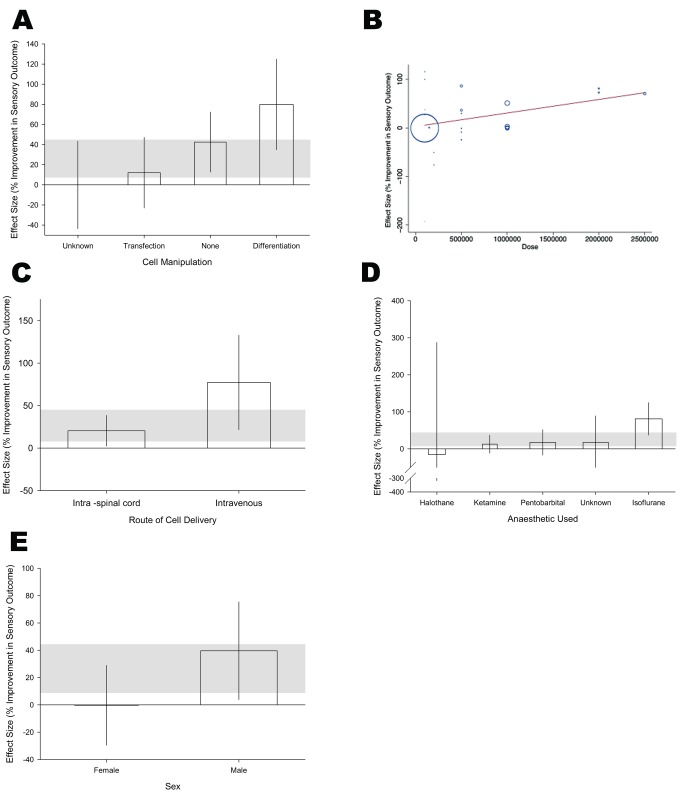
Study characteristics that account for heterogeneity in sensory score. (A) Type of manipulation of stem cells prior to implantation, (B) dose–response relationship, (C) route of stem cell delivery, (D) anaesthetic used, and (E) sex. The shaded grey bar represents the 95% confidence limits of the global estimate. The vertical error bars represent the 95% confidence intervals for the individual estimates.

Of the five study characteristics accounting for a significant proportion of the between-study heterogeneity, the type of manipulation in culture had the largest effect (adjusted R^2^ = 61.3%, *p*<0.005). Prior differentiation was associated with larger effect sizes, while transfection was associated with smaller effects (Figure 5A). The number of cells administered had a clear dose–response effect (adjusted R^2^ = 31.7%, *p*<0.02; Figure 5B). Studies that delivered cells intravenously were associated with significantly larger effects than studies transplanting the cells directly into the lesion area of the spinal cord (adjusted R^2^ = 19.2%, *p*<0.05) (Figure 5C).

As with the motor score subanalysis, the anaesthetic agent had a large effect (adjusted R^2^ = 42.8%, *p*<0.05). The use of isoflurane to induce anaesthesia in three individual comparisons was associated with substantial additional benefit compared to other methods of anaesthesia (Figure 5D). All studies assessed sensory outcome in either all male or all female cohorts, with studies using female animals appearing to offer no benefit (Figure 5E; adjusted R^2^ = 21.5%, *p*<0.05).

## Discussion

Systematic review and meta-analysis have helped identify biases within clinical trials [49], providing an impetus to improve standards [50]. This approach offers similar benefits for animal studies [28],[41],[51] by describing the impact of biological and experimental factors on reported efficacy in a systematic and transparent summary of all available data. This allows judgement of the extent to which conclusions are at risk of bias [52]. In this study we apply these techniques to provide a detailed systematic analysis of the animal literature describing stem-cell–based therapies in spinal cord injury.

Overall, treatment with allogeneic stem cells improves both motor and sensory outcome after spinal cord injury by around 25%, but with important differences between the two datasets. Because of the amount of data, conclusions relating to motor outcome (5,628 animals) are probably more robust than those relating to sensory outcomes (473 animals). For both outcomes there was a broad range of experimental approaches, reflected in the high levels of heterogeneity seen. This is typical for systematic reviews in animal studies and validates our choice of a random effects model, and our summary estimates should be considered as the average efficacy rather than the best estimate of a single “true” efficacy. Interestingly, improvement in sensory outcome seems to be sensitive to differences in factors relating to treatment (i.e., stem cell biology), while motor outcome appears to be more sensitive to factors relating to the lesion and the outcome measure used, and to be less dependent on the biological features of the stem cells used.

Evidence supporting a dose–response relationship for sensory outcome suggests the presence of a biologically plausible effect. We observed that prior differentiation of the implanted cells was associated with larger effects. Where the influence of cell differentiation was formally studied, a relationship with outcome was observed [53]. This suggests that optimal efficacy might be seen when cells have some lineage specificity but before final cell type commitment has occurred. For sensory outcome, studies where cells were delivered intravenously, rather than directly into the injured spinal cord, were associated with significantly larger effects. This suggests either that systemic changes may mediate the effects of stem cells or that local implantation may create additional injury that masks the benefit provided by stem cells.

We did not see a dose–response relationship for motor outcomes, even where we limited our analysis to a more homogenous subset of experiments. It may be that there is no dose–response effect or that the doses used in these experiments were all large enough to generate maximal responses. Where dose response was formally studied the authors found increasing benefit from doses as low as 10,000 implanted cells [54], and the median number of implanted cells in comparisons reporting motor outcomes was 250,000.

Immunosuppression with cyclosporine A was associated with increased efficacy in a systematic review of stem cells in focal cerebral ischaemia [28], and it is therefore interesting that in spinal cord injury both cyclosporine A and FK506 are associated with reduced efficacy. This suggests that any beneficial effect of immunosupressants in promoting the survival of transplanted cells is outweighed by other factors, such as effects on stem cell biology or intrinsic repair mechanisms. Unfortunately, because of the univariate nature of our analyses we are unable to determine a “benefit–risk ratio” for the use of immunosuppression. However, there are studies that indicate that bone-marrow–derived stem cells are able to produce compartmentalised inflammatory lesions [55],[56]. The mechanisms behind this observation are not understood, yet there are rising concerns that unwanted inflammatory-driven side effects, such as neuropathic pain, might limit the “usefulness” of gained motor function.

For motor outcome, the neurobehavioural test used (Figure 3A) accounted for most of the observed heterogeneity. The BBB locomotor rating scale was used in 70% of animals. In the more focussed analysis of rat allogeneic, midthoracic impact injury, using BBB as an outcome, studies that used other behavioural tests in addition to the BBB reported smaller effect sizes for the BBB. This may be a manifestation of outcome reporting bias; if the outcome on the BBB is smaller than expected, investigators might also report the outcome on other tests where the effect was larger; if the effect measured using the BBB was considered “sufficient,” there might be less motivation also to report outcomes using other measures, particularly if these were smaller than seen using the BBB.

Overall, there was no improvement in motor outcome where this was assessed using the staircase or mouse hind limb motor score tests. However, these accounted for a small proportion of the overall dataset, and so these results should be interpreted with caution.

Efficacy was strongly associated with both the location of and the methodology used to create the injury. The largest effect was seen with lower thoracic and lumbar lesions and when the spinal cord was lesioned by hemisection or transsection rather than contusion or compression.

The use of isoflurane anaesthesia at SCI induction was associated with substantial improvement in sensory outcome; in the overall motor analysis, there was no effect, but in the more homogenous restricted analysis, isoflurane was again associated with substantially larger effects. Again, this contrasts with findings in focal cerebral ischaemia and suggests that, despite interest in a general paradigm of “neuroprotection,” these conditions are in certain respects biologically very different. However, these findings are based on a small number of individual comparisons and should be interpreted with caution.

The sex of the experimental animal accounted for a large proportion of the observed heterogeneity in both the sensory and motor analyses. For the motor analyses, this seems to be the influence of abnormally high effect sizes reported in studies where either the sex of the animals used was not reported or where “both sexes” were used. For sensory outcome, studies using male animals led to significantly higher estimates of effect with no clear benefit detected in female animals.

Thirty percent of animals in our dataset were treated with stem cells at the time of injury. Although this may be helpful in the biological assessment of stem cell therapies, it is of limited clinical relevance. The time of administration, although important with regard to translation to a clinical setting, had no significant impact on the effects reported. This appears to be somewhat unlikely, and our findings may mask different efficacies of different stem cell approaches at different times—those with more neuroprotective characteristics perhaps being more effective when given early, and those with more influence on neuroregeneration and repair being more effective when given late.

We found that the prevalence of reporting of randomisation and blinded assessment of outcome was higher than that reported in the modelling of other neurological disorders, suggesting more rigour in the conduct of these studies [39]–[42]. Other markers of internal validity, such as sample size calculations, were rarely reported (Table 1). The lack of an *a priori* sample size calculation increases the risk that group sizes were increased during the experiment, in light of analysis showing borderline nonsignificant results; this is an important potential source of bias. It is of course possible that some authors had taken measures to reduce bias but did not report them; this underlines the importance of reporting guidelines [57],[58].

For the larger motor dataset, both publication bias (Figure 2B) and failure to report blinding (Figure 3H) were both associated with a significant overestimation of overall effect size; there was no apparent impact of a failure to report randomisation. In the Egger regression (Figure 2B) removal of the two most extreme data points did not change the interpretation that publication bias was present (not shown).

Stratification of the data to determine the effect of the above facets of experimentation is desirable. However, no publication randomised, blinded assessment of outcome, concealed allocation, and performed a sample size calculation and only 20 individual comparisons came from papers describing three of the four. Therefore, we subanalysed the 25% of the motor dataset that reported having both randomised and blinded.

In this subanalysis the characteristics of the animal model still have more impact than the type of cells implanted. However, there were differences, but the reductionist approach of this subanalysis does raise the possibility that these might be false positives due to loss of power. The type of cell culture medium and type of cell manipulation prior to implantation appear to have an impact, but it should be noted that in both cases it is the experiments where the precise conditions are “unknown” that report the greatest effect. There is no obvious biological explanation for this. It may be that a failure to report such details is a surrogate indication that such work is generally of lower quality, and therefore at greater risk of bias.

Immunosuppression is no longer identified as accounting for a significant proportion of the heterogeneity. However, the effect size in cyclosporine-A–treated animals (mean, 24.3; 95% CI, 13.2–35.3) is the same as in animals where no immune suppression was used (mean, 24.9; 95% CI, 18.3–31.6). This appears to confirm that immune suppression offers no advantage in experiments using allogeneic implants to treat SCI.

Intriguingly, in the subanalysis a dose–response relationship does emerge. As the mean number of cells implanted is 6.3×10^5^ rather than 7.4×10^8^ in the full motor dataset, this is consistent with the hypothesis that such an effect was previously masked by a ceiling effect.

### 

#### Limitations of our approach

Firstly, we were only able to include data from studies in the public domain and—for motor outcome at least—there is evidence of a publication bias in favour of studies with large effect sizes. Further, we found some evidence (in the motor BBB subanalysis) consistent with selective reporting of outcomes within individual publications. The true effect sizes are therefore likely to be lower than reported here. Secondly, for both study quality and study design features, we relied on published information. Where relevant information was not available (the sex of a cohort of animals, or the taking of measures to reduce bias), we have either analysed these as not known or inferred that things that were not reported did not occur. Thirdly, we present a series of univariate analyses; multivariate meta-regression or stepwise partitioning of heterogeneity might provide more robust insights, but these techniques are not well established. Similarly, for continuous variables, the meta-regressions reported here assumed a linear relationship between the independent and dependent variables, and this is likely that this represents an oversimplification, at least for some independent variables. Fourthly, we have observed the experiments of others rather than conducted experiments of our own, and this observational research should be considered as hypothesis generating only. Finally, we limited our analysis to neurobehavioural outcomes; the greater benefit seen in hemisected and transsected lesions compared with compressive of contusional injuries may have important histological correlates, and this is worthy of further exploration.

In conclusion, stem cells appear to have substantial efficacy in animal models of traumatic SCI. Effects on sensory outcome appear more dependent on facets of stem cell biology: motor outcome appears to be more dependent on features of the animal modelling and the outcome scale used.

## Methods

The study protocol is available at www.camarades.info/index_files/Protocols.html. A completed PRISMA checklist and flow diagram for this systematic literature review can be found in Text S1.

### Definitions

We define a “publication” as a discrete piece of work (including abstracts); each publication may report data from a number of experiments. Each experiment may describe outcome in a number of different experimental cohorts, and the contrast between outcomes in a single treatment cohort with that in a control cohort we define as an “individual comparison.” We define “nesting” as combining the effect sizes from different functional outcomes measured in the same cohort of animals to give a single summary estimate of effect in that individual comparison (a nested individual comparison).

### Systematic Review

Using prespecified inclusion and exclusion criteria we identified all publications reporting relevant experiments (see below) by searching (December 2011) three electronic databases (PubMed, EMBASE, and ISI Web of Science) using the search strategy “(stem cell OR stem OR haematopoietic OR mesenchymal) AND (spinal cord injury OR hemisection OR contusion injury OR dorsal column injury OR complete transection OR corticospinal tract injury),” with search results limited to those indexed as describing animal experiments.

### Inclusion and Exclusion Criteria

Two investigators (A.A. and E.S.) independently reviewed retrieved publications. We included experiments where functional outcome in a group of animals exposed to traumatic spinal cord injury and treated with allogeneic or autologous stem cells was compared with functional outcome in a control group of animals. We excluded individual comparisons that did not report (or where we could not calculate) the number of animals, the mean outcome, or its variance in each group. We excluded experiments where interventions such as growth factors were used to mobilise endogenous stem cells or where nontraumatic models of spinal cord injury were used.

### Data Extraction

From each individual comparison we extracted data for reported outcomes. This included extraction of mean and variance data from each cohort exposed to an intervention (controls and active therapy) and from sham cohorts of normal (unlesioned and untreated) animals, and by imputation where the performance of a normal animal could be imputed from the description of the scoring scale. Stem cells were characterised as “autologous” where cells were extracted from an animal, might be manipulated in some way, then returned to the same animal; or “allogeneic” where embryonic or adult cells derived from a different animal were administered to a recipient animal. Where a publication reported more than one experiment, or where an experiment reported more than one individual comparison (for instance, increasing numbers of stem cells transplanted), we considered these separately and extracted data for each, correcting the weighting of these studies in meta-analysis to reflect the number of experimental groups served by each control group. Where different functional outcomes were reported in a single cohort of animals, we combined these outcomes using fixed effects meta-analysis (nesting), to give a summary estimate of functional outcome in that cohort, described here as a comparison. Where a test involved exposing the animal to increasing intensities of the same stimulus (for instance, in allodynia testing), we used data for the median intensity. For sensory tests, only data for stimulation distal to the lesion were included. Where functional outcome was measured at different times, we extracted data for the last time point reported.

Study quality was assessed using a checklist adapted from good laboratory practice guidelines for *in vivo* stroke modelling [59] and the CAMARADES quality checklist [60]. The checklist comprised (i) publication in a peer-reviewed journal, (ii) statements describing control of temperature, (iii) randomisation to treatment group, (iv) allocation concealment, (v) blinded assessment of outcome, (vi) avoidance of anaesthetics with known marked intrinsic neuroprotective properties, (vii) sample size calculation, (viii) compliance with animal welfare regulations, and (ix) whether the authors declared any potential conflict of interest.

### Analysis

For each individual comparison, we calculate a normalised effect size [normalised mean difference) as the percentage improvement (“+” sign) or worsening (“−” sign) of outcome in the treatment group using the following formula:
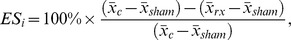
where 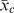 and 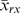 are the mean reported outcomes in the control and treatment group, respectively, and 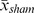 is the mean outcome for a normal (unlesioned and untreated) animal. In this calculation, the score achieved by the sham animals acts as the “fixed zero value” or baseline allowing the difference between the sham and treatment groups to be expressed as a ratio. This ratio takes into account differences in the “direction” of individual neurobehavioural scales.

Its corresponding standard error was calculated using:
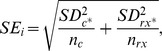
where 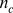 refers to the number of animals in the control group and 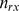 refers to the number of animals in the treatment group. 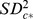 and 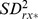 are the normalised standard deviations for the control and treatment group, respectively. These were calculated using the formulae:
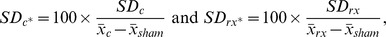
where *SD_c_* and *SD_rx_* are the reported standard deviation for the control and treatment group, respectively.

We then used DerSimonian and Laird random effects weighted mean difference meta-analysis to calculate a summary estimate of effect size; results are presented as the percentage improvement in outcome and its 95% confidence intervals. The variability of the outcomes assessed is presented as the heterogeneity statistic (χ^2^) with *n*−1 degrees of freedom.

The analysis was stratified according to (i) the approach to stem cell therapy (allogeneic, autologous, embryonic, source of cells, *ex vivo* manipulation), (ii) biological factors (number of cells, time and route of administration, time of assessment of outcome), (iii) aspects of study design (anaesthesia, species of animal, immunosuppression, model and severity of spinal cord injury), and (iv) elements of study quality.

The extent to which study design characteristics explained differences between studies was assessed using meta-regression with the *metareg* function of STATA/SE10, and the significance level was set at *p*<0.05. The meta-regression was univariate rather than multivariate; and we calculated adjusted R^2^ values (a measure of how much residual heterogeneity is explained by the model) to explain the proportion of the observed variability in the observed effect size for a group of experiments explained by variation in the independent variable in question [61].

We sought evidence of publication bias using a funnel plot, Egger regression, and Trim and Fill [62]. A detailed description of the statistical methods used for meta-analysis and meta-regression can be found in [63].

## Supporting Information

Table S1 **Included studies.** First author, publication year, stem cell used, species of host animal, number of animals, number of cells, time of treatment in relation to injury, anaesthetic used, type of injury, route of delivery, and outcome measure reported for studies included in the review.
(DOCX)

Table S2 Quality of included studies/reporting of (1) publication in a peer-reviewed journal, (2) statement describing control of temperature, (3) randomisation to treatment group, (4) allocation concealment, (5) blinded assessment of outcome, (6) avoidance of anaesthetic with known marked intrinsic neuroprotective properties, (7) sample size calculation, (8) compliance with animal welfare regulations, and (9) statement of any potential conflict of interest.
(DOCX)

Text S1 PRISMA 2009 Checklist.
(DOC)

## Abbreviations

AD: Alzheimer's disease
BBB: Basso Beattie and Bresnehan
BMS: Basso mouse scale
CAMARADES: Collaborative Approach to Meta-Analysis and Review of Animal Data from Experimental Studies
CI: Confidence interval
CNS: Central nervous system
EAE: Experimental autoimmune encephalomyelitis
FCI: Focal cerebral ischaemia
SCI: Spinal cord injury; PD, Parkinson's disease
χ^2^: Chi-squared, heterogeneity

## References

[1] Thomas ED, Lochte HL Jr, Lu WC, Ferrebee JW (1957) Intravenous infusion of bone marrow in patients receiving radiation and chemotherapy. N Engl J Med 257: 491–496. 13464965 10.1056/NEJM195709122571102

[2] Gratwohl A, Baldomero H, Aljurf M, Pasquini MC, Bouzas LF, et al (2010) Hematopoietic stem cell transplantation. JAMA 303: 1617–1624. 20424252 10.1001/jama.2010.491 PMC3219875

[3] Liu H, Kim Y, Sharkis S, Marchionni L, Jang Y-Y (2011) In vivo liver regeneration potential of human induced pluripotent stem cells from diverse origins. Sci Transl Med 3: 82ra39. 10.1126/scitranslmed.3002376 PMC3305909 21562231

[4] Rama P, Matuska S, Paganoni G, Spinelli A, De Luca M, et al (2010) Limbal stem-cell therapy and long-term corneal regeneration. N Engl J Med 363: 147–155. 20573916 10.1056/NEJMoa0905955

[5] Yao L, Li Z-r, Su W-r, Li Y-p, Lin M-l, et al (2012) Role of mesenchymal stem cells on cornea wound healing induced by acute alkali burn. PLoS ONE 7: e30842. doi:10.1371/journal.pone.0030842. 22363499 10.1371/journal.pone.0030842 PMC3281878

[6] Gong Z, Niklason LE (2008) Small-diameter human vessel wall engineered from bone marrow-derived mesenchymal stem cells (hMSCs). FASEB J 22: 1635–1648. 18199698 10.1096/fj.07-087924 PMC2605790

[7] Zwi-Dantsis L, Huber I, Habib M, Winterstern A, Gepstein A, et al (2012) Derivation and cardiomyocyte differentiation of induced pluripotent stem cells from heart failure patients. Eur Heart J 34 21: 1575–1586. 22621821 10.1093/eurheartj/ehs096

[8] Ott HC, Matthiesen TS, Goh S-K, Black LD, Kren SM, et al (2008) Perfusion-decellularized matrix: using nature's platform to engineer a bioartificial heart. Nat Med 14: 213–221. 18193059 10.1038/nm1684

[9] Qian L, Huang Y, Spencer CI, Foley A, Vedantham V, et al (2012) In vivo reprogramming of murine cardiac fibroblasts into induced cardiomyocytes. Nature 485: 593–598. 22522929 10.1038/nature11044 PMC3369107

[10] Ott HC, Clippinger B, Conrad C, Schuetz C, Pomerantseva I, et al (2010) Regeneration and orthotopic transplantation of a bioartificial lung. Nat Med 16: 927–933. 20628374 10.1038/nm.2193

[11] Song JJ, Guyette JP, Gilpin SE, Gonzalez G, Vacanti JP, et al (2013) Regeneration and experimental orthotopic transplantation of a bioengineered kidney. Nat Med 19 5: 646–651. 23584091 10.1038/nm.3154 PMC3650107

[12] Denham M, Parish CL, Leaw B, Wright J, Reid CA, et al (2012) Neurons derived from human embryonic stem cells extend long-distance axonal projections through growth along host white matter tracts after intra-cerebral transplantation. Front Cell Neurosci 6: 11. 22470319 10.3389/fncel.2012.00011 PMC3311135

[13] Ideguchi M, Palmer TD, Recht LD, Weimann JM (2010) Murine embryonic stem cell-derived pyramidal neurons integrate into the cerebral cortex and appropriately project axons to subcortical targets. J Neurosci 30: 894–904. 20089898 10.1523/JNEUROSCI.4318-09.2010 PMC3842463

[14] Steinbeck JA, Koch P, Derouiche A, Brüstle O (2012) Human embryonic stem cell-derived neurons establish region-specific, long-range projections in the adult brain. Cell Mol Life Sci 69: 461–470. 21779868 10.1007/s00018-011-0759-6 PMC3256316

[15] Kaigler D, Pagni G, Park C, Braun T, Holman L, et al (2012) Stem cell therapy for craniofacial bone regeneration: a randomized, controlled, feasibility trial. Cell Transplant 22 5: 767–777. 10.3727/096368912X652968 PMC4100608 22776413

[16] Tan J, Wu W, Xu X, Liao L, Zheng F, et al (2012) Induction therapy with autologous mesenchymal stem cells in living-related kidney transplants: a randomized controlled trial. JAMA 307: 1169–1177. 22436957 10.1001/jama.2012.316

[17] Leventhal J, Abecassis M, Miller J, Gallon L, Ravindra K, et al (2012) Chimerism and tolerance without GVHD or engraftment syndrome in HLA-mismatched combined kidney and hematopoietic stem cell transplantation. Sci Transl Med 4: 124ra128. 10.1126/scitranslmed.3003509 PMC3610325 22399264

[18] Jia Z, Jiao C, Zhao S, Li X, Ren X, et al (2012) Immunomodulatory effects of mesenchymal stem cells in a rat corneal allograft rejection model. Exp Eye Res 102: 44–49. 22800963 10.1016/j.exer.2012.06.008

[19] Kuo Y-R, Chen C-C, Goto S, Huang Y-T, Wang C-T, et al (2012) Immunomodulatory effects of bone marrow-derived mesenchymal stem cells in a swine hemi-facial allotransplantation model. PLoS ONE 7: e35459. doi:10.1371/journal.pone.0035459. 22558153 10.1371/journal.pone.0035459 PMC3338845

[20] McGill TJ, Cottam B, Lu B, Wang S, Girman S, et al (2012) Transplantation of human central nervous system stem cells–neuroprotection in retinal degeneration. Eur J Neurosci 35 3: 468–477. 22277045 10.1111/j.1460-9568.2011.07970.x

[21] Tamaki SJ, Jacobs Y, Dohse M, Capela A, Cooper JD, et al (2009) Neuroprotection of host cells by human central nervous system stem cells in a mouse model of infantile neuronal ceroid lipofuscinosis. Cell Stem Cell 5: 310–319. 19733542 10.1016/j.stem.2009.05.022

[22] Cabanes C, Bonilla S, Tabares L, Martínez S (2007) Neuroprotective effect of adult hematopoietic stem cells in a mouse model of motoneuron degeneration. Neurobiol Dis 26: 408–418. 17337196 10.1016/j.nbd.2007.01.008

[23] Su H, Zhang W, Guo J, Guo A, Yuan Q, et al (2009) Neural progenitor cells enhance the survival and axonal regeneration of injured motoneurons after transplantation into the avulsed ventral horn of adult rats. J Neurotrauma 26: 67–80. 19196181 10.1089/neu.2008.0656

[24] Yasuhara T, Matsukawa N, Hara K, Yu G, Xu L, et al (2006) Transplantation of human neural stem cells exerts neuroprotection in a rat model of Parkinson's disease. J Neurosci 26: 12497–12511. 17135412 10.1523/JNEUROSCI.3719-06.2006 PMC6674904

[25] Aharonowiz M, Einstein O, Fainstein N, Lassmann H, Reubinoff B, et al (2008) Neuroprotective effect of transplanted human embryonic stem cell-derived neural precursors in an animal model of multiple sclerosis. PLoS ONE 3: e3145. doi:10.1371/journal.pone.0003145. 18773082 10.1371/journal.pone.0003145 PMC2522282

[26] Morando S, Vigo T, Esposito M, Casazza S, Novi G, et al (2012) The therapeutic effect of mesenchymal stem cell transplantation in experimental autoimmune encephalomyelitis is mediated by peripheral and central mechanisms. Stem Cell Res Ther 3: 1–7. 22277374 10.1186/scrt94 PMC3340547

[27] Capone C, Frigerio S, Fumagalli S, Gelati M, Principato M-C, et al (2007) Neurosphere-derived cells exert a neuroprotective action by changing the ischemic microenvironment. PLoS ONE 2: e373. doi:10.1371/journal.pone.0000373. 17440609 10.1371/journal.pone.0000373 PMC1847533

[28] Lees JS, Sena ES, Egan KJ, Antonic A, Koblar SA, et al (2012) Stem cell-based therapy for experimental stroke: A systematic review and meta-analysis. Int J Stroke 7: 582–588. 22687044 10.1111/j.1747-4949.2012.00797.x

[29] Yang C, Zhou L, Gao X, Chen B, Tu J, et al (2011) Neuroprotective effects of bone marrow stem cells overexpressing glial cell line-derived neurotrophic factor on rats with intracerebral hemorrhage and neurons exposed to hypoxia/reoxygenation. Neurosurgery 68: 691. 21311297 10.1227/NEU.0b013e3182098a8a

[30] Chung JY, Kim W, Im W, Yoo DY, Choi JH, et al (2012) Neuroprotective effects of adipose-derived stem cells against ischemic neuronal damage in the rabbit spinal cord. J Neurol Sci 317 1–2: 40–46. 22475376 10.1016/j.jns.2012.02.035

[31] Novikova LN, Brohlin M, Kingham PJ, Novikov LN, Wiberg M (2011) Neuroprotective and growth-promoting effects of bone marrow stromal cells after cervical spinal cord injury in adult rats. Cytotherapy 13: 873–887. 21521004 10.3109/14653249.2011.574116

[32] Carvalho K, Cunha R, Vialle E, Osiecki R, Moreira G, et al. Functional outcome of bone marrow stem cells (CD45^+^/CD34^−^) after cell therapy in acute spinal cord injury: In Exercise training and in sedentary rats; 2008. Elsevier. pp. 847–849. 10.1016/j.transproceed.2008.02.055 18455034

[33] Basso DM, Beattie MS, Bresnahan JC (1995) A sensitive and reliable locomotor rating scale for open field testing in rats. J Neurotrauma 12: 1–21. 7783230 10.1089/neu.1995.12.1

[34] Basso DM, Fisher LC, Anderson AJ, Jakeman LB, Mctigue DM, et al (2006) Basso Mouse Scale for locomotion detects differences in recovery after spinal cord injury in five common mouse strains. J Neurotrauma 23: 635–659. 16689667 10.1089/neu.2006.23.635

[35] Tarlov I, Klinger H (1954) Spinal cord compression studiesII. Time limits for recovery after acute compression in dogs. AMA Arch Neurol Psychiatry 71: 271–290. 13123590

[36] Marshall JF (1982) Sensorimotor disturbances in the aging rodent. J Gerontol 37: 548–554. 7096925 10.1093/geronj/37.5.548

[37] Simiand J, Keane P, Morre M (1984) The staircase test in mice: a simple and efficient procedure for primary screening of anxiolytic agents. Psychopharmacology 84: 48–53. 6149594 10.1007/BF00432023

[38] Koshizuka S, Okada S, Okawa A, Koda M, Murasawa M, et al (2004) Transplanted hematopoietic stem cells from bone marrow differentiate into neural lineage cells and promote functional recovery after spinal cord injury in mice. J Neuropathol Exp Neurol 63: 64–72. 14748562 10.1093/jnen/63.1.64

[39] Egan KJ, Sena ES, Vesterinen HM, Macleod MR (2011) Making the most of animal data–improving the prospect of success in pragmatic trials in the neurosciences. Trials 12: A102.

[40] Rooke ED, Vesterinen HM, Sena ES, Egan KJ, Macleod MR (2011) Dopamine agonists in animal models of Parkinson's disease: a systematic review and meta-analysis. Parkinsonism Relat Disord 17: 313–320. 21376651 10.1016/j.parkreldis.2011.02.010

[41] Sena E, van der Worp HB, Howells D, Macleod M (2007) How can we improve the pre-clinical development of drugs for stroke? Trend Neurosci 30: 433–439. 17765332 10.1016/j.tins.2007.06.009

[42] Vesterinen HM, Sena ES, Williams A, Chandran S, Macleod MR (2010) Improving the translational hit of experimental treatments in multiple sclerosis. Multiple Sclerosis 16: 1044. 20685763 10.1177/1352458510379612

[43] Dietrich WD, Atkins CM, Bramlett HM (2009) Protection in animal models of brain and spinal cord injury with mild to moderate hypothermia. J Neurotrauma 26: 301–312. 19245308 10.1089/neu.2008.0806 PMC2848835

[44] Kwon BK, Mann C, Sohn HM, Hilibrand AS, Phillips FM, et al (2008) Hypothermia for spinal cord injury. Spine J 8: 859. 18329959 10.1016/j.spinee.2007.12.006

[45] Batchelor PE, Kerr NF, Gatt AM, Aleksoska E, Cox SF, et al (2010) Hypothermia prior to decompression: Buying time for treatment of acute spinal cord injury. J Neurotrauma 27: 1357–1368. 20504158 10.1089/neu.2010.1360

[46] van der Worp HB, Sena ES, Donnan GA, Howells DW, Macleod MR (2007) Hypothermia in animal models of acute ischaemic stroke: a systematic review and meta-analysis. Brain 130: 3063–3074. 17478443 10.1093/brain/awm083

[47] Bernard SA, Gray TW, Buist MD, Jones BM, Silvester W, et al (2002) Treatment of comatose survivors of out-of-hospital cardiac arrest with induced hypothermia. New Engl J Med 346: 557–563. 11856794 10.1056/NEJMoa003289

[48] Shankaran S, Laptook AR, Ehrenkranz RA, Tyson JE, McDonald SA, et al (2005) Whole-body hypothermia for neonates with hypoxic–ischemic encephalopathy. New Eng J Med 353: 1574–1584. 16221780 10.1056/NEJMcps050929

[49] Begg C, Cho M, Eastwood S, Horton R, Moher D, et al (1996) Improving the quality of reporting of randomized controlled trials. JAMA 12: 33–35. 10.1001/jama.276.8.637 8773637

[50] Kane RL, Wang J, Garrard J (2007) Reporting in randomized clinical trials improved after adoption of the CONSORT statement. J Clin Epidemiol 60: 241. 17292017 10.1016/j.jclinepi.2006.06.016

[51] Crossley NA, Sena E, Goehler J, Horn J, van der Worp B, et al (2008) Empirical evidence of bias in the design of experimental stroke studies: a metaepidemiologic approach. Stroke 39: 929–934. 18239164 10.1161/STROKEAHA.107.498725

[52] Van Der Worp HB, Macleod MR, Kollmar R (2010) Therapeutic hypothermia for acute ischemic stroke: ready to start large randomized trials? J Cereb Blood Flow Metab 30: 1079–1093. 20354545 10.1038/jcbfm.2010.44 PMC2949207

[53] Hofstetter CP, Holmström NA, Lilja JA, Schweinhardt P, Hao J, et al (2005) Allodynia limits the usefulness of intraspinal neural stem cell grafts; directed differentiation improves outcome. Nat Neurosci 8: 346–353. 15711542 10.1038/nn1405

[54] Zhao P, Feng S, Wang Y, P Z, Feng SQ, et al (2010) Effect of different concentration of human umbilical cord mesenchymal stem cells in experimental spinal cord injury in rats. Zhongguo Jiaoxing Waike Zazhi/Orthopedic Journal of China 18: 1817–1825.

[55] Snyder EY (2011) The risk of putting something where it does not belong: mesenchymal stem cells produce masses in the brain. Exp Neurol 230: 75. 21420402 10.1016/j.expneurol.2011.03.012

[56] Grigoriadis N, Lourbopoulos A, Lagoudaki R, Frischer J-M, Polyzoidou E, et al (2011) Variable behavior and complications of autologous bone marrow mesenchymal stem cells transplanted in experimental autoimmune encephalomyelitis. Exp Neurol 230: 78–89. 21440544 10.1016/j.expneurol.2011.02.021

[57] Kilkenny C, Browne WJ, Cuthill IC, Emerson M, Altman DG (2010) Improving bioscience research reporting: the ARRIVE guidelines for reporting animal research. Plos Biol 8: e1000412. doi:10.1371/journal.pbio.1000412. 20613859 10.1371/journal.pbio.1000412 PMC2893951

[58] Landis SC, Amara SG, Asadullah K, Austin CP, Blumenstein R, et al (2012) A call for transparent reporting to optimize the predictive value of preclinical research. Nature 490: 187–191. 23060188 10.1038/nature11556 PMC3511845

[59] Macleod MR, Fisher M, O'Collins V, Sena ES, Dirnagl U, et al (2009) Good laboratory practice: preventing introduction of bias at the bench. Stroke 40: e50–e52. 18703798 10.1161/STROKEAHA.108.525386 PMC2729492

[60] Macleod MR, O'Collins T, Howells DW, Donnan GA (2004) Pooling of animal experimental data reveals influence of study design and publication bias. Stroke 35: 1203–1208. 15060322 10.1161/01.STR.0000125719.25853.20

[61] Harbord RM, Higgins JP (2008) Meta-regression in Stata. Stata J 8: 493–519.

[62] Sena ES, van der Worp HB, Bath PM, Howells DW, Macleod MR (2010) Publication bias in reports of animal stroke studies leads to major overstatement of efficacy. Plos Biol 8: e1000344–e1000352. doi:10.1371/journal.pbio.1000344. 20361022 10.1371/journal.pbio.1000344 PMC2846857

[63] Vesterinen H, Sena E, Egan K, Hirst T, Currie G, Antonic A, et al (2013) Meta-analysis of data from animal studies: a practical guide. J Neurosci Methods. doi:pii: S0165-0270(13)00321-X. 10.1016/j.jneumeth.2013.09.010. [Epub ahead of print]. 10.1016/j.jneumeth.2013.09.010 24099992

