# Supplementary material for: Stem Cell Transplantation in Traumatic Spinal Cord Injury: A Systematic Review and Meta-Analysis of Animal Studies
Source: PLoS Biol. 2013 Dec 17;11(12):e1001738. doi: 10.1371/journal.pbio.1001738 (PMC3866091; doi:10.1371/journal.pbio.1001738)
Supplement: Table S1 — Included studies. First author, publication year, stem cell used, species of host animal, number of animals, number of cells, time of treatment in relation to injury, anaesthetic used, type of injury, route of delivery, and outcome measure reported for studies included in the review. (DOCX) [file pbio.1001738.s001.docx]

***Table S1: Study Characteristics***

**Publication Stem Cell Species # Animals # Cells Time of Anaesthetic Type of Route of Outcome Admin (mins) Injury Delivery Measure(s)**

Abematsu,M 2010 [1] mouse NSC Mouse 73 1x10^6^ Cells 10080 Pentobarbital Contusion ISpinalCord Motor

Abramas,M 2009 [2] rat MSC Rat 80 1.5x10^5^ Cells 10080 Unknown Contusion ISpinalCord Sensory & Motor

Alexanian,A 2011 [3] human MSC Rat 27 1x10^5^ Cells 10080 Ketamine Contusion ISpinalCord Motor

Alexanian,A 2010 [4] mouse MSC Rat 30 2.5x10^5^ Cells 10080 Ketamine Contusion ISpinalCord Motor

Alexanian,A 2011 [5] Human NPCs Rat 120 2.5x10^4^ Cells 10080 Ketamine Contusion ISpinalCord Motor

Amemori,T 2010 [6] rat MSC Rat 57 3x10^5^ Cells 10080 Isoflurane Compression ISpinalCord Motor

Amoh,Y 2008 [7] mouse hair follicle Mouse 14 Unknown 86400 Tribromoethanol Transection ISpinalCord Motor stem cells

Ankeny,D 2004 [8] rat BMSC Rat 11 3x10^5^ Cells 2880 Unknown Contusion ISpinalCord Motor

Arboleda,S 2011 [9] Rat ASC Rat 35 5x10^5^ Cells 10080 Isoflurane Compression ISpinalCord Motor

Bi,X 2008 [10] rat BMSC Rat 24 1x10^6^ Cells 10080 Chloral Hydrate Contusion ISpinalCord Motor

Boido,M 2009 [11] mouse MSC Mouse 45 1x10^5^ Cells 20160 Isoflurane Hemisection ISpinalCord Motor mouse NPC Mouse 53 1x10^5^ Cells 20160 Isoflurane Hemisection ISpinalCord Motor

Boido,M 2011 [12] mouse NPC Mouse 185 1x10^5^ Cells 0 Unknown Compression ISpinalCord Motor & Senory

Bottai,D 2008 [13] mouse NSC Mouse 45 1x10^6^ Cells 30 Unknown Contusion IVenous Motor

Bozkurt,G 2010 [14] rat spinal cord Rat 27 1x10^6^ Cells 30240 Halothane Compression ISpinalCord Motor NSPCs

Cao,Q 2005 [15] rat glial-restricted Rat 33 4x10^5^ Cells 12960 Pentobarbital Contusion ISpinalCord Motor precursors

Cao,Q 2010 [16] Rat OPC Rat 26 1x10^5^ Cells 11520 Pentobarbital Contusion ISpinalCord Motor

Carvalho,K 2008 [17] rat BMSC Rat 36 5x10^6^ Cells 2880 Ketamine Contusion ISpinalCord Motor

**Publication Stem Cell Species # Animals # Cells Time of Anaesthetic Type of Route of Outcome Admin (mins) Injury Delivery Measure(s)**

Chen,G 2010 [18] rat spinal cord Rat 36 2x10^5^ Cells 0 Chloral Hydrate Hemisection ISpinalCord Motor NSPCs

Chen,X 2011 [19] rat BMSC Rat 22 1x10^5^ Cells 0 Pentobarbital Transection ISpinalCord Motor

Chen,Y 2010 [20] rat BMSC Rat 40 3x10^5^ Cells 5 Chloral Hydrate Compression ISpinalCord Motor

Chiba,Y 2009 [21] rat BMSC Rat 14 7x10^4^ Cells 10080 Isoflurane Contusion ISpinalCord Motor

Cho,S 2008 [22] human MPCs Rat 22 2.5x10^5^ Cells 10080 Pentobarbital Contusion ISpinalCord Motor human MSC Rat 6 2.5x10^5^ Cells 10080 Pentobarbital Contusion ISpinalCord Motor human NPCs Rat 18 2.5x10^5^ Cells 10080 Pentobarbital Contusion ISpinalCord Motor

Cizkova,D 2006 [23] human MSC Rat 30 1x10^6^ Cells 10080 Halothane Compression IVenous Motor

Cizkova,D 2011 [24] rat MSC Rat 44 5x10^5^-1.5x10^6^ Cells 4320-12960 Halothane Compression ISpinalCord Motor

Cui,Y 2011 [25] mouse ESC Mouse 112 1x10^4^ Cells 1440 Unknown Compression ISpinalCord Motor

Cummings,B 2005 [26] human fetal NSC Mouse 84 1.87x10^4^ Cells 12960 Tribromoethanol Contusion ISpinalCord Motor

Dasari,V 2007 [27] rat BMSC Rat 54 2.5x10^5^ Cells 10080 Ketamine Contusion ISpinalCord Motor

Dasari,V 2008 [28] human UCBC Rat 87 2.5x106 Cells 10080 Ketamine Contusion ISpinalCord Motor & Sensory

Dasari,V 2007 [29] human UCBC Rat 27 2.5x10^5^ Cells 10080 Ketamine Contusion ISpinalCord Motor

Davies,J 2006 [30] rat glial-restricted Rat 12 1.8x10^5^ Cells 0 Ketamine Contusion ISpinalCord Motor

precursors

Davies,S 2011 [31] hGPC Rat 12 1.8x10^5^ Cells 0 Ketamine Transection ISpinalCord Motor hGDA Rat 31 1.8x10^5^ Cells 0 Ketamine Transection ISpinalCord Motor

de Almeida,L 2011 [32] Human dental Mouse 32 8x10^5^ Cells 10080-40320 Ketamine Compression ISpinalCord Motor

pulp cells

Deng,X 2010 [33] human UCBC Rat 80 6x10^3^ Cells 0 Unknown Transection ISpinalCord Motor

Deng,Y 2008 [34] human BMSC Rat 60 2.5x10^5^ Cells 30 Chloral Hydrate Contusion ISpinalCord Motor **Publication Stem Cell Species # Animals # Cells Time of Anaesthetic Type of Route of Outcome Admin (mins) Injury Delivery Measure(s)**

Diao,Y 2009 [35] rat BMSC Rat 12 5x10^5^ Cells 0 Unknown Hemisection ISpinalCord Motor

Ding,Y 2009 [36] rat BMSC Rat 20 5x10^4^ Cells 10080 Pentobarbital Transection ISpinalCord Motor

Du,B 2011 [37] rat NSC Rat 34 Unknown 0 Pentobarbital Transection ISpinalCord Motor

Erceg,S 2010 [38] human ESC Rat 84 75x10^4^ Cells 0 Pentobarbital Transection ISpinalCord Motor

Fan,D 2011 [39] rat BMSC Rat 24 5x10^7^ Cells 0 Unknown Hemisection ISpinalCord Motor

Fang,K 2010 [40] human MSC Rat 19 2x10^5^ Cells 10080 Pentobarbital Contusion ISpinalCord Motor

Gorio,A 2004 [41] Rat Dermis Rat 80 1.5x10^5^ Cells 30-10080 Chloral Hydrate Contusion ISpinalCord Motor Stem Cells

Guo,J 2007 [42] rat NSC Rat 200 5x10^6^ Cells 0 Pentobarbital Transection ISpinalCord Motor

Guo,Y 2011 [43] Human Umbilical Rat 36 1x10^6^ Cells 0 Chloral Hydrate Transection ISpinalCord Motor MSC

Hains,B 2002 [44] RN46A-B14 Rat 210 1x10^6^ Cells 0 Pentobarbital Hemisection ISpinalCord Motor & Sensory cell line

Hains,B 2003 [45] RN46A-B14 Rat 135 1x10^6^ Cells 40320 Pentobarbital Hemisection ISpinalCord Motor cell line

Han,S 2006 [46] rat NSC Rat 45 1.5x10^6^ Cells 12960 amobarbital Transection ISpinalCord Motor

Hasegawa,K 2004 [47] rat NSC Rat 32 8x10^5^ Cells 0 Pentobarbital Contusion ISpinalCord Motor

Hatami,M 2009 [48] human ESC Rat 30 2.5x10^5^ Cells 8640 Ketamine Hemisection ISpinalCord Motor

Himes,B 2006 [49] human BMSC Rat 49 5x10^5^-1x10^6^ Cells 10080 Ketamine Contusion ISpinalCord Motor

Hirofumi,S 2011 [50] rat BMSC Rat 18 1x10^5^ Cells 1440 Pentobarbital Contusion ISpinalCord Motor

Hofstetter,C 2005 [51] rat NSC Rat 250 1x10^5^ Cells 10080 Halothane Contusion ISpinalCord Motor & Sensory

Hofstetter,H 2002 [52] rat BMSC Rat 38 1.5x10^5^ Cells 0-10080 Halothane Contusion ISpinalCord Motor

Howard,M 2005 [53] mouse ESC Rat 21 1x10^5^ Cells 12960 Ketamine Contusion ISpinalCord Motor

**Publication Stem Cell Species # Animals # Cells Time of Anaesthetic Type of Route of Outcome Admin (mins) Injury Delivery Measure(s)**

Hu,S 2010 [54] human UCBC Rat 24 4x10^5^ Cells 0 Pentobarbital Compression ISpinalCord Motor

Hwang,D 2011 [55] hNSC line F3 Rat 100 5x10^4^-5x10^5^ Cells 0 Chloral Hydrate Hemisection ISpinalCord Motor

Ide,C 2010 [56] rat BMSC Rat 112 5x10^5^ Cells 20160 Pentobarbital Contusion ISpinalCord Motor

Iwanami,A 2005 [57] Human NPCs Marmoset 20 1x10^6^ Cells 12960 Ketamine Contusion ISpinalCord Motor

Jin,Y 2011 [58] hGDA Rat 36 1x10^5^ Cells 12960 Ketamine Contusion ISpinalCord Motor & Sensory hGPC Rat 45 1x10^5^ Cells 12960 Ketamine Contusion ISpinalCord Motor & Sensory

Jing,W 2008 [59] rat BMSC Rat 36 1x10^6^ Cells 10080 Unknown Contusion ISpinalCord Motor

Joghataei,M 2010 [60] rat BMSC Rat 32 3x10^5^ Cells 10080 Ketamine Contusion ISpinalCord Motor

Kamada,T 2010 [61] human BMSC Rat 12 2x10^6^ Cells 10080 Halothane Contusion ISpinalCord Motor

Kamei,N 2010 [62] mouse BMSC Mouse 20 1x10^5^ Cells 0 Tribromoethanol Compression ISpinalCord Motor mouse BMSC Mouse 20 1x10^5^ Cells 0 Tribromoethanol Compression ISpinalCord Motor

Kang,K 2011 [63] rat BMSC Rat 15 2x10^6^ Cells 0 Ketamine Hemisection ISpinalCord Motor

Karimi- 2006 [64] mouse NPC Rat 16 3.5x10^5^ Cells 336 Halothane Compression ISpinalCord Motor Abdolrezaee,S

Keirstead,H 2005 [65] human ESC Rat 22 2.5x10^5^ Cells 10080 Ketamine Contusion ISpinalCord Motor

Kim,D 2010 [66] mouse ESC Rat 116 5x10^5^ Cells 20160 Isoflurane Hemisection ISpinalCord Sensory

Koda,M 2005 [67] mouse BMSC Mouse 15 3x10^4^ Cells 10080 Unknown Compression ISpinalCord Motor mouse hemotopietic Mouse 15 3x10^4^ Cells 10080 Unknown Compression ISpinalCord Motor stem cell

Koshizuka,S 2004 [68] mouse BMSC Mouse 19 3x10^4^ Cells 10080 Halothane Compression ISpinalCord Motor

Lebedev,S 2010 [69] rat NSC Rat 53 7.5x10^5^ Cells 0 Ketamine Compression ISpinalCord Motor

Lee,K 2007 [70] human MSC Rat 116 5x10^5^ Cells 10080 Pentobarbital Contusion ISpinalCord Motor & Sensory

**Publication Stem Cell Species # Animals # Cells Time of Anaesthetic Type of Route of Outcome Admin (mins) Injury Delivery Measure(s)**

Lee,K 2005 [71] Rat OPC Rat 80 5x10^5^ Cells 10080 Pentobarbital Contusion ISpinalCord Motor

Lee,K 2011 [72] human BMSC Rat 24 1x10^5^ Cells 10080 Pentobarbital Contusion ISpinalCord Motor

Li,C 2004 [73] rat NSC Rat 20 5x10^5^ Cells 0 Pentobarbital Transection ISpinalCord Motor

Li,H 2004 [74] human UCBC Rat 20 8x10^4^ Cells 0 Pentobarbital Hemisection ISpinalCord Motor

Li,H 2010 [75] rat BMSC Rat 75 1x10^6^ - 5x10^6^ Cells 10080 Pentobarbital Compression ISpinalCord Motor

Li,W 2006 [76] rat NSC Rat 32 6x10^5^ Cells 0 Unknown Transection ISpinalCord Motor

Li,Y 2011 [77] mouse NSC Rat 25 6x10^5^ Cells 0-10080 Chloral Hydrate Transection ISpinalCord Motor

Liang,P 2006 [78] human NSC Rat 30 5x10^4^ Cells 0 Ketamine Transection ISpinalCord Motor

Lin,J 2010 [79] rat BMSC Rat 72 1x10^6^ Cells 4320 Unknown Contusion ISpinalCord Motor

Liu,W 2011 [80] rat BMSC Rat 96 2x10^5^ Cells 10 Unknown Compression ISpinalCord Motor

Liu,Y 2011 [81] rat MSC Rat 24 1x10^5^ Cells 10080 Chloral Hydrate Contusion ISpinalCord Motor

Lu,P 2005 [82] rat BMSC Rat 36 2x10^5^ Cells 0 Ketamine Hemisection ISpinalCord Motor & Sensory

Macias,M 2006 [83] mouse NPC Rat 207 1x10^5^ Cells 11520 Ketamine Contusion ISpinalCord Motor & Sensory

Marques 2010 [84] mouse ESC Mouse 68 8x10^5^ Cells 10 Ketamine Compression ISpinalCord Motor

Matsuda,R 2009 [85] mouse ESC Mouse 30 1.5x10^4^ Cells 11520 Pentobarbital Contusion ISpinalCord Motor

McDonald,J 1999 [86] mouse ESC Rat 34 1x10^6^ Cells 12960 Pentobarbital Contusion ISpinalCord Motor

Mcmahon,S 2010 [87] mouse NSC Rat 56 1x10^5^ Cells 10080 Ketamine Contusion ISpinalCord Motor

Meng,X 2008 [88] rat NSC Rat 48 1x10^5^ Cells 10080 Chloral Hydrate Contusion ISpinalCord Motor

Mitsui,T 2005 [89] rat NPC Rat 20 1x10^6^ Cells 12960 Ketamine Contusion ISpinalCord Motor

Moreno- 2009 [90] Rat OPC Rat 13 1x10^6^ Cells 11340 Isoflurane Contusion ISpinalCord Motor Manzano,V rat spinal cord Rat 20 1x10^6^ Cells 11340 Isoflurane Contusion ISpinalCord Motor ependymal stem cells

**Publication Stem Cell Species # Animals # Cells Time of Anaesthetic Type of Route of Outcome Admin (mins) Injury Delivery Measure(s)**

Nakamura,M 2005 [91] rat NSC Rat 78 2x10^6^ Cells 0 Hypothermia Hemisection ISpinalCord Motor

Neuhuber,B 2005 [92] human MSC Rat 280 5x10^5^ Cells 0 Ketamine Hemisection ISpinalCord Motor & Sensory

Neuhuber,B 2008 [93] rat NPC Rat 30 1x10^6^-2x10^6^ Cells 216 Ketamine Contusion ISpinalCord Motor

Nishio,Y 2006 [94] human UCBC Rat 19 5.5x10^6^ Cells 10080 Halothane Contusion IPeritoneal Motor

Nomura,H 2008 [95] Rat Brain NSPC Rat 17 3x10^6^ Cells 0 Unknown Transection ISpinalCord Motor rat spinal cord Rat 18 3x10^6^ Cells 0 Unknown Transection ISpinalCord Motor NSPCs

Nori,S 2011 [96] Human NPCs Mouse 60 5x10^5^ Cells 12960 Ketamine Contusion ISpinalCord Motor

Ohta,M 2004 [97] rat BMSC Rat 32 5x10^6^ Cells 0 Pentobarbital Contusion ISpinalCord Motor

Olson,H 2009 [98] rat NSC Rat 25 4.76x10^5^ Cells 0 Ketamine Transection ISpinalCord Motor

Osaka,M 2010 [99] rat MSC Rat 188 1x10^6^ Cells 360-40320 Ketamine Contusion IVenous Motor

Pallini,R 2005 [100] mouse NSC Mouse 49 1x10^5^ Cells 0 Ketamine Transection ISpinalCord Motor

Pan,H 2008 [101] rat NSC Rat 33 5x10^6^ Cells 10080 Isoflurane Transection ISpinalCord Motor

Parr,A 2007 [102] rat spinal cord Rat 17 Unknown 0 Halothane Compression ISpinalCord Motor NSPCs

Parr,A 2008 [103] rat BMSC Rat 10 2.5x10^4^ Cells 12960 Halothane Compression ISpinalCord Motor rat spinal cord Rat 10 2.5x10^4^ Cells 12960 Halothane Compression ISpinalCord Motor ependymal stem cells

Parr,A 2008 [104] rat BMSC Rat 79 2x10^5^ Cells 0 Halothane Compression ISpinalCord Motor Rat NSPCs Rat 39 2x10^5^ Cells 12960 Halothane Compression ISpinalCord Motor

Pedram,M 2010 [105] rat BMSC Rat 23 1x10^6^ -2x10^6^ Cells 10080 Unknown Compression ISpinalCord Motor

Pu,Y 2007 [106] rat NSC Rat 30 5x10^5^ Cells 0 Unknown Transection Unknown Motor

Pu,Y 2007 [107] rat NSC Rat 45 Unknown 0 Unknown Hemisection ISpinalCord Motor

**Publication Stem Cell Species # Animals # Cells Time of Anaesthetic Type of Route of Outcome Admin (mins) Injury Delivery Measure(s)**

Qin,M 2008 [108] ESC Rat 36 4x10^5^ Cells 12960 Pentobarbital Transection ISpinalCord Motor

Rossi,S 2010 [109] human ESC Rat 202 1x10^5^ Cells 10080 Ketamine Contusion ISpinalCord Motor

Saporta,S 2003 [110] human UCBC Rat 18 4x10^3^ Cells 1440-7200 Isoflurane Compression ISpinalCord Motor

Setoguchi,T 2004 [111] mouse NPC Mouse 39 1x10^5^ Cells 11520 Pentobarbital Compression ISpinalCord Motor

Shang,A 2011 [112] Human Umbilical Rat 120 1x10^6^ Cells 10080 Pentobarbital Compression ISpinalCord Motor MSC

Shen,J 2009 [113] rat MSC Rat 36 1x10^5^ Cells 10080 Chloral Hydrate Contusion ISpinalCord Motor

Sheth,R 2008 [114] human BMSC Rat 76 6x10^5^ Cells 10080 Ketamine Contusion ISpinalCord Motor

Someya,Y 2008 [115] rat BMSC Rat 48 5x10^5^ Cells 10080 Unknown Contusion ISpinalCord Motor

Sun,Y 2003 [116] rat NSC Rat 30 4x10^4^ Cells 20160 amobarbital Compression ISpinalCord Motor

Takenaga,M 2007 [117] mouse ESC Rat 24 5x10^6^ Cells 12960 Unknown Compression SubCut Motor

Takeuchi,H 2007 [118] human NSC Mouse 14 1x10^6^ Cells 10080 Unknown Compression IVenous Motor

Tarasenko,Y 2007 [119] human fetal NSC Rat 27 2x10^5^ Cells 0 Pentobarbital Contusion ISpinalCord Motor

Teng,Y 2002 [120] murine NSC Rat 49 1x10^5^ Cells 0 Chloral Hydrate Hemisection ISpinalCord Motor murine NSC Rat 49 1x10^5^ Cells 0 Chloral Hydrate Hemisection ISpinalCord Motor

Urdzikova,L 2006 [121] rat BMSC Rat 45 2x10^6^ Cells 10080 Isoflurane Compression IVenous Motor & Sensory rat MSC Rat 45 2x10^6^ Cells 10080 Isoflurane Compression IVenous Motor & Sensory

Wang,C 2011 [122] rat MSC Rat 18 1x10^6^ Cells 4320 Unknown Contusion ISpinalCord Motor

Wang,D 2010 [123] rat BMSC Rat 39 5x10^5^ Cells 0 Pentobarbital Hemisection ISpinalCord Motor rat BMSC Rat 39 5x10^5^ Cells 0 Pentobarbital Hemisection ISpinalCord Motor

Wang,D 2010 [124] rat BMSC Rat 39 1x10^10^ Cells 360 Ketamine Hemisection Ivenous Motor

Wang,D 2010 [125] rat BMSC Rabbit 44 2x10^6^ Cells 4320 Ketamine Contusion IVenous Motor

Wang,G 2010 [126] rat NSC Rat 24 1x10^7^ Cells 10080 Chloral Hydrate Hemisection ISpinalCord Motor

**Publication Stem Cell Species # Animals # Cells Time of Anaesthetic Type of Route of Outcome Admin (mins) Injury Delivery Measure(s)**

Wang,T 2007 [127] rat MSC Rat 42 5x10^6^ Cells 1440-30240 phenobarbital Transection ISpinalCord Motor

Wang,T 2010 [128] rat DMSCs Rat 48 2x10^6^ Cells 10080 Pentobarbital Transection ISpinalCord Motor

Watanabe,K 2003 [129] rat spinal cord Rat 23 5x10^5^ Cells 216 Unknown Contusion ISpinalCord Motor NSPCs rat forebrain NSPCs Rat 25 5x10^5^ Cells 216 Unknown Contusion ISpinalCord Motor

Wu,S 2003 [130] rat BMSC Rat 18 1x10^6^ Cells 0 Pentobarbital Contusion ISpinalCord Motor

Wu,W 2011 [131] rat BMSC Rat 60 5x10^5^ Cells 10080 Chloral Hydrate Contusion ISpinalCord Motor

Wu,Z 2006 [132] Human AEC Rat 14 1x10^5^ Cells 0 Chloral Hydrate Transection ISpinalCord Motor

Xu,G 2003 [133] G3 line Rat 20 5x10^5^ Cells 12960 Chloral Hydrate Contusion ISpinalCord Motor

Xu,G 2004 [134] Human NPCs Rat 20 1x10^5^ Cells 12960 Chloral Hydrate Contusion ISpinalCord Motor

Xu,Z 2011 [135] rat BMSC Rat 168 1.25x10^5^ Cells 80640 Pentobarbital Contusion ISpinalCord Motor

Yan,Q 2011 [136] rat MSC Rat 20 5x10^5^ Cells 0 Pentobarbital Transection ISpinalCord Motor

Yang,C 2008 [137] Human Umbilical Rat 28 1x10^6^ Cells 0 Halothane Transection ISpinalCord Motor MSC

Yang,J 2007 [138] mouse ESC Mouse 19 9x10^4^ Cells 0 Unknown Hemisection ISpinalCord Motor

Yano,S 2006 [139] rat BMSC Rat 11 7.5x10^4^ Cells 10080 Isoflurane Contusion ISpinalCord Motor

Yin,G 2006 [140] human NSC Rat 48 5x10^3^ Cells 14400 Pentobarbital Transection ISpinalCord Motor

Yoshihara,H 2006 [141] rat MSC Rat 60 1x10^6^ Cells 12960 Ketamine Contusion ISpinalCord Motor

Yoshihara,T 2007 [142] rat BMSC Rat 16 5x10^6^ Cells 60 Pentobarbital Contusion ISpinalCord Motor

Yu,D 2011 [143] rat BMSC Rat 66 3x10^5^ Cells 30 Chloral Hydrate Compression ISpinalCord Motor

Zhang,H 2007 [144] human UCBC Rat 72 1x106 Cells 7200 Chloral Hydrate Compression IVenous Motor

**Publication Stem Cell Species # Animals # Cells Time of Anaesthetic Type of Route of Outcome Admin (mins) Injury Delivery Measure(s)**

Zhang,H 2009 [145] Rat SVZ-NS Rat 66 3x10^5^ Cells 10080 Pentobarbital Contusion ISpinalCord Motor Rat AD-NS Rat 66 3x10^5^ Cells 10080 Pentobarbital Contusion ISpinalCord Motor Rat BM-NS Rat 66 3x10^5^ Cells 10080 Pentobarbital Contusion ISpinalCord Motor

Zhang,H 2010 [146] rat MSC Rat 44 1x10^6^ Cells 10080 Chloral Hydrate Contusion ISpinalCord Motor

Zhang,J 2010 [147] rat BMSC Rat 100 1x10^7^ Cells 10080 Phencylidine Hemisection ISpinalCord Motor Hydrochloride

Zhang,L 2007 [148] mouse NPC Mouse 30 2x10^5^ Cells 12960 Chloral Hydrate Transection ISpinalCord Motor

Zhang,W 2010 [149] rat BMSC Rat 36 5x10^4^-5x10^5^ Cells 0 Pentobarbital Transection ISpinalCord Motor

Zhang,X 2007 [150] rat NSC Rat 11 2.5x10^5^ Cells 0 Pentobarbital Transection ISpinalCord Motor

Zhang,Z 2010 [151] rat BMSC Rat 20 1x10^6^ Cells 10080 Pentobarbital Contusion ISpinalCord Motor

Zhao,H 2004 [152] human BMSC Rat 30 5x10^4^ Cells 0 Pentobarbital Hemisection ISpinalCord Motor

Zhao,H 2004 [152] Human umbilical Rat 30 5x10^4^ Cells 0 Pentobarbital Hemisection ISpinalCord Motor

cord cells CD34+

Zhao,P 2010 [153] human UCBC Rat 76 1x10^4^ - 1x10^6^ Cells 10080 Chloral Hydrate Contusion ISpinalCord Motor

Zhilai,Z 2011 [154] rat BMSC Rat 32 2x10^5^ Cells 10080 Pentobarbital Contusion ISpinalCord Motor

Zhou,Z 2011 [155] rat BMSC Rat 32 2x10^5^ Cells 10080 Unknown Contusion ISpinalCord Motor

Ziv,Y 2006 [156] murine neural Mouse 91 5x10^5^ Cells 10080 Unknown Contusion ICerebVentr Motor

precursor cells

Reference List

1. Abematsu M, Tsujimura K, Yamano M, Saito M, Kohno K, Kohyama J, Namihira M, Komiya S, Nakashima K (2010) Neurons derived from transplanted neural stem cells restore disrupted neuronal circuitry in a mouse model of spinal cord injury. Journal of Clinical Investigation 120: 3255-3266.

2. Abrams MB, Dominguez C, Pernold K, Regera R, Wiesenfeld-Hallin Z, Olson L, Prockop D (2009) Multipotent mesenchymal stromal cells attenuate chronic inflammation and injury-induced sensitivity to mechanical stimuli in experimental spinal cord injury. Restorative Neurology and Neuroscience 27: 307-321.

3. Alexanian AR, Fehlings MG, Zhang Z, Maiman DJ (2011) Transplanted Neurally Modified Bone Marrow-Derived Mesenchymal Stem Cells Promote Tissue Protection and Locomotor Recovery in Spinal Cord Injured Rats. Neurorehabilitation and Neural Repair 25: 873-880.

4. Alexanian AR, Kwok WM, Pravdic D, Maiman DJ, Fehlings MG (2010) Survival of neurally induced mesenchymal cells may determine degree of motor recovery in injured spinal cord rats. Restor Neurol Neurosci 28: 761-767.

5. Alexanian AR, Svendsen CN, Crowe MJ, Kurpad SN (2011) Transplantation of human glial-restricted neural precursors into injured spinal cord promotes functional and sensory recovery without causing allodynia. Cytotherapy 13: 61-68.

6. Amemori T, Jendelova P, Ruzickova K, Arboleda D, Sykova E (2010) Co-transplantation of olfactory ensheathing glia and mesenchymal stromal cells does not have synergistic effects after spinal cord injury in the rat. Cytotherapy 12: 212-225.

7. Amoh Y, Li LN, Katsuoka K, Hoffman RM (2008) Multipotent hair follicle stem cells promote repair of spinal cord injury and recovery of walking function. Cell Cycle 7: 1865-1869.

8. Ankeny DP, McTigue DM, Jakeman LB (2004) Bone marrow transplants provide tissue protection and directional guidance for axons after contusive spinal cord injury in rats. Experimental Neurology 190: 17-31.

9. Arboleda D, Forostyak S, Jendelova P, Marekova D, Amemori T, Pivonkova H, Masinova K, Sykova E (2011) Transplantation of Predifferentiated Adipose-Derived Stromal Cells for the Treatment of Spinal Cord Injury. Cellular and Molecular Neurobiology 31: 1113-1122.

10. Bi XB, Deng YB, Gan DH, Wang YZ (2008) Salvianolic acid B promotes survival of transplanted mesenchymal stem cells in spinal cord-injured rats. Acta Pharmacol Sin 29: 169-176.

11. Boido M, Rupa R, Garbossa D, Fontanella M, Ducati A, Vercelli A (2009) Embryonic and adult stem cells promote raphespinal axon outgrowth and improve functional outcome following spinal hemisection in mice. European Journal of Neuroscience 30: 833-846.

12. Boido M, Garbossa D, Vercelli A (2011) Early graft of neural precursors in spinal cord compression reduces glial cyst and improves function. J Neurosurg Spine 15: 97-106.

13. Bottai D, Madaschi L, Di Giulio AM, Gorio A (2008) Viability-dependent promoting action of adult neural precursors in spinal cord injury. Molecular Medicine 14: 634-644.

14. Bozkurt G, Mothe AJ, Zahir T, Kim H, Shoichet MS, Tator CH (2010) Chitosan channels containing spinal cord-derived stem/progenitor cells for repair of subacute spinal cord injury in the rat. Neurosurgery 67: 1733-1744.

15. Cao Q, Xu XM, Devries WH, Enzmann GU, Ping P, Tsoulfas P, Wood PM, Bunge MB, Whittemore SR (2005) Functional recovery in traumatic spinal cord injury after transplantation of multineurotrophin-expressing glial-restricted precursor cells. J Neurosci 25: 6947-6957.

16. Cao Q, He Q, Wang Y, Cheng X, Howard RM, Zhang Y, DeVries WH, Shields CB, Magnuson DSK, Xu XM, Kim DH, Whittemore SR (2010) Transplantation of Ciliary Neurotrophic Factor-Expressing Adult Oligodendrocyte Precursor Cells Promotes Remyelination and Functional Recovery after Spinal Cord Injury. Journal of Neuroscience 30: 2989-3001.

17. Carvalho KA, Cunha RC, Vialle EN, Osiecki R, Moreira GH, Simeoni RB, Francisco JC, Guarita-Souza LC, Oliveira L, Zocche L, Olandoski M (2008) Functional outcome of bone marrow stem cells (CD45(+)/CD34(-)) after cell therapy in acute spinal cord injury: in exercise training and in sedentary rats. Transplant Proc 40: 847-849.

18. Chen G, Hu YR, Wan H, Xia L, Li JH, Yang F, Qu X, Wang SG, Wang ZC (2010) Functional recovery following traumatic spinal cord injury mediated by a unique polymer scaffold seeded with neural stem cells and Schwann cells. Chin Med J (Engl) 123: 2424-2431.

19. Chen X, Yang Y, Yao J, Lin W, Li Y, Chen Y, Gao Y, Yang Y, Gu X, Wang X (2011) Bone marrow stromal cells-loaded chitosan conduits promote repair of complete transection injury in rat spinal cord. Journal of Materials Science-Materials in Medicine 22: 2347-2356.

20. Chen Y, Wang S, Geng B, Wang C, Zhao L, Ma Y, Xia Y, Liu W (2010) [Experimental study on adenosine triphosphate combining bone marrow mesenchymal stem cells transplantation in treatment of spinal cord injury in rats]. Zhongguo Xiu Fu Chong Jian Wai Ke Za Zhi 24: 1233-1238.

21. Chiba Y, Kuroda S, Maruichi K, Osanai T, Hokari M, Yano S, Shichinohe H, Hida K, Iwasaki Y (2009) Transplanted Bone Marrow Stromal Cells Promote Axonal Regeneration and Improve Motor Function in a Rat Spinal Cord Injury Model. Neurosurgery 64: 991-999.

22. Cho SR, Yang MS, Yim SH, Park JH, Lee JE, Eom YW, Jang IK, Kim HE, Park JS, Kim HO, Lee BH, Park CI, Kim YJ (2008) Neurally induced umbilical cord blood cells modestly repair injured spinal cords. Neuroreport 19: 1259-1263.

23. Cizkova D, Rosocha J, Vanicky I, Jergova S, Cizek M (2006) Transplants of human mesenchymal stem cells improve functional recovery after spinal cord injury in the rat. Cellular and Molecular Neurobiology 26: 1165-1178.

24. Cizkova D, Novotna I, Slovinska L, Vanicky I, Jergova S, Rosocha J, Radonak J (2011) Repetitive Intrathecal Catheter Delivery of Bone Marrow Mesenchymal Stromal Cells Improves Functional Recovery in a Rat Model of Contusive Spinal Cord Injury. Journal of Neurotrauma 28: 1951-1961.

25. Cui YF, Xu JC, Hargus G, Jakovcevski I, Schachner M, Bernreuther C (2011) Embryonic Stem Cell-Derived L1 Overexpressing Neural Aggregates Enhance Recovery after Spinal Cord Injury in Mice. PLoS One 6.

26. Cummings BJ, Uchida N, Tamaki SJ, Salazar DL, Hooshmand M, Summers R, Gage FH, Anderson AJ (2005) Human neural stem cells differentiate and promote locomotor recovery in spinal cord-injured mice. Proceedings of the National Academy of Sciences of the United States of America 102: 14069-14074.

27. Dasari VR, Spomar DG, Cady C, Gujrati M, Rao JS, Dinh DH (2007) Mesenchymal stem cells from rat bone marrow downregulate caspase-3-mediated apoptotic pathway after spinal cord injury in rats. Neurochemical Research 32: 2080-2093.

28. Dasari VR, Spomar DG, Li L, Gujrati M, Rao JS, Dinh DH (2008) Umbilical cord blood stem cell mediated downregulation of Fas improves functional recovery of rats after spinal cord injury. Neurochemical Research 33: 134-149.

29. Dasari VR, Spomar DG, Gondi CS, Sloffer CA, Saving KL, Gujrati M, Rao JS, Dinh DH (2007) Axonal remyelination by cord blood stem cells after spinal cord injury. Journal of Neurotrauma 24: 391-410.

30. Davies JE, Huang C, Proschel C, Noble M, Mayer-Proschel M, Davies SJ (2006) Astrocytes derived from glial-restricted precursors promote spinal cord repair. J Biol 5: 7.

31. Davies SJ, Shih CH, Noble M, Mayer-Proschel M, Davies JE, Proschel C (2011) Transplantation of specific human astrocytes promotes functional recovery after spinal cord injury. PLoS One 6: e17328.

32. de Almeida FM, Marques SA, Ramalho BdS, Rodrigues RF, Cadilhe DV, Furtado D, Kerkis I, Pereira LV, Rehen SK, Blanco Martinez AM (2011) Human Dental Pulp Cells: A New Source of Cell Therapy in a Mouse Model of Compressive Spinal Cord Injury. Journal of Neurotrauma 28: 1939-1949.

33. Deng Xy, Zhou Rp, Lu Kw, Jin Dd (2010) Lithium chloride combined with human umbilical cord blood mesenchymal stem cell transplantation for treatment of spinal cord injury in rats. Nan fang yi ke da xue xue bao = Journal of Southern Medical University 30: 2436-2439.

34. Deng YB, Liu Y, Zhu WB, Bi XB, Wang YZ, Ye MH, Zhou GQ (2008) The co-transplantation of human bone marrow stromal cells and embryo olfactory ensheathing cells as a new approach to treat spinal cord injury in a rat model. Cytotherapy 10: 551-564.

35. Diao Yn, Ma Ll, Meng Fb, Li Ht, Pang Xn (2010) Transplantation of Bone Marrow Mesenchymal Stem Cells Promote Functional Recovery of the Injured Rats Spinal Cord. Journal of China Medical University 39: 7-9.

36. Ding Y, Yan Q, Ruan JW, Zhang YQ, Li WJ, Zhang YJ, Li Y, Dong HX, Zeng YS (2009) Electro-acupuncture promotes survival, differentiation of the bone marrow mesenchymal stem cells as well as functional recovery in the spinal cord-transected rats. Bmc Neuroscience 10.

37. Du BL, Xiong Y, Zeng CG, He LM, Zhang W, Quan DP, Wu JL, Li Y, Zeng YS (2011) Transplantation of artificial neural construct partly improved spinal tissue repair and functional recovery in rats with spinal cord transection. Brain Res 1400: 87-98.

38. Erceg S, Ronaghi M, Oria M, Garcia Rosello M, Perez Arago MA, Gomez Lopez M, Radojevic I, Moreno-Manzano V, Rodriguez-Jimenez FJ, Shanker Bhattacharya S, Cordoba J, Stojkovic M (2010) Transplanted Oligodendrocytes and Motoneuron Progenitors Generated from Human Embryonic Stem Cells Promote Locomotor Recovery After Spinal Cord Transection. Stem Cells 28: 1541-1549.

39. Fan Dy, Wang P, Liu R, Chen Yb (2011) Repair of Spinal Cord Injury in Rats by Transplantation with Bone Marrow Mesenchymal Stem Cells and Stimulus of Direct Current. Chinese Journal of Biologicals 24: 1028-1032.

40. Fang KM, Chen JK, Hung SC, Chen MC, Wu YT, Wu TJ, Lin HI, Chen CH, Cheng H, Yang CS, Tzeng SF (2010) Effects of combinatorial treatment with pituitary adenylate cyclase activating peptide and human mesenchymal stem cells on spinal cord tissue repair. PLoS One 5: e15299.

41. Gorio A, Torrente Y, Madaschi L, Di Stefano AB, Pisati F, Marchesi C, Belicchi M, Di Giulio AM, Bresolin N (2004) Fate of autologous dermal stem cells transplanted into the spinal cord after traumatic injury (TSCI). Neuroscience 125: 179-189.

42. Guo JS, Zeng YS, Li HB, Huang WL, Liu RY, Li XB, Ding Y, Wu LZ, Cai DZ (2007) Cotransplant of neural stem cells and NT-3 gene modified Schwann cells promote the recovery of transected spinal cord injury. Spinal Cord 45: 15-24.

43. Guo YW, Ke YQ, Li M, Cai YQ, Jiang XD, Zhang SZ, Zhang WM, Duan CZ (2011) Human Umbilical Cord-Derived Schwann-Like Cell Transplantation Combined with Neurotrophin-3 Administration in Dyskinesia of Rats with Spinal Cord Injury. Neurochemical Research 36: 783-792.

44. Hains BC, Yucra JA, Eaton MJ, Hulsebosch CE (2002) Intralesion transplantation of serotonergic precursors enhances locomotor recovery but has no effect on development of chronic central pain following hemisection injury in rats. Neurosci Lett 324: 222-226.

45. Hains BC, Johnson KM, Eaton MJ, Willis WD, Hulsebosch CE (2003) Serotonergic neural precursor cell grafts attenuate bilateral hyperexcitability of dorsal horn neurons after spinal hemisection in rat. Neuroscience 116: 1097-1110.

46. Han S, Ling S, Zhu X, Liang X (2006) Effect of Annexin II on the repair of spinal cord injury of the rats. Jiepou Xuebao 37: 627-632.

47. Hasegawa K, Chang YW, Li HD, Berlin Y, Ikeda O, Kane-Goldsmith N, Grumet M (2005) Embryonic radial glia bridge spinal cord lesions and promote functional recovery following spinal cord injury. Experimental Neurology 193: 394-410.

48. Hatami M, Mehrjardi NZ, Kiani S, Hemmesi K, Azizi H, Shahverdi A, Baharvand H (2009) Human embryonic stem cell-derived neural precursor transplants in collagen scaffolds promote recovery in injured rat spinal cord. Cytotherapy 11: 618-630.

49. Himes BT, Neuhuber B, Coleman C, Kushner R, Swanger SA, Kopen GC, Wagner J, Shumsky JS, Fischer I (2006) Recovery of function following grafting of human bone marrow-derived stromal cells into the injured spinal cord. Neurorehabilitation and Neural Repair 20: 278-296.

50. Sasaki H, Tanaka N, Nakanishi K, Nishida K, Hamasaki T, Yamada K, Ochi M (2011) Therapeutic Effects With Magnetic Targeting of Bone Marrow Stromal Cells in a Rat Spinal Cord Injury Model. Spine 36: 933-938.

51. Hofstetter CP, Holmstrom NAV, Lilja JA, Schweinhardt P, Hao J, Spenger C, Wiesenfeld-Hallin Z, Kurpad SN, Frisen J, Olson L (2005) Allodynia limits the usefulness of intraspinal neural stem cell grafts; directed differentiation improves outcome. Nature Neuroscience 8: 346-353.

52. Hofstetter CP, Schwarz EJ, Hess D, Widenfalk J, El Manira A, Prockop DJ, Olson L (2002) Marrow stromal cells form guiding strands in the injured spinal cord and promote recovery. Proceedings of the National Academy of Sciences of the United States of America 99: 2199-2204.

53. Howard MJ, Liu S, Schottler F, Snider BJ, Jacquin MF (2005) Transplantation of apoptosis-resistant embryonic stem cells into the injured rat spinal cord. Somatosensory and Motor Research 22: 37-44.

54. Hu SL, Luo HS, Li JT, Xia YZ, Li L, Zhang LJ, Meng H, Cui GY, Chen Z, Wu N, Lin JK, Zhu G, Feng H (2010) Functional recovery in acute traumatic spinal cord injury after transplantation of human umbilical cord mesenchymal stem cells. Critical Care Medicine 38: 2181-2189.

55. Hwang DH, Kim HM, Kang YM, Joo IS, Cho CS, Yoon BW, Kim SU, Kim BG (2011) Combination of multifaceted strategies to maximize the therapeutic benefits of neural stem cell transplantation for spinal cord repair. Cell Transplantation 20: 1361-1379.

56. Ide C, Nakai Y, Nakano N, Seo TB, Yamada Y, Endo K, Noda T, Saito F, Suzuki Y, Fukushima M, Nakatani T (2010) Bone marrow stromal cell transplantation for treatment of sub-acute spinal cord injury in the rat. Brain Research 1332: 32-47.

57. Iwanami A, Kaneko S, Nakamura M, Kanemura Y, Mori H, Kobayashi S, Yamasaki M, Momoshima S, Ishii H, Ando K, Tanioka Y, Tamaoki N, Nomura T, Toyama Y, Okano H (2005) Transplantation of human neural stem cells for spinal cord injury in primates. Journal of Neuroscience Research 80: 182-190.

58. Jin Y, Neuhuber B, Singh A, Bouyer J, Lepore A, Bonner J, Himes T, Campanelli JT, Fischer I (2011) Transplantation of human glial restricted progenitors and derived astrocytes into a contusion model of spinal cord injury. J Neurotrauma 28: 579-594.

59. Jing W-L, Yan F-X, Zuo Y-Z (2008) Comparison of various mesenchymal stem cell transplatation pathways for treatment of spinal cord injury in rats. [Chinese]. Journal of Clinical Rehabilitative Tissue Engineering Research 12: 10045-10049.

60. Joghataei MT, Bakhtiari M, Pourheydar B, Mehdizadeh M, Faghihi A, Mehraein F, Behnam B, Pirhajati V (2010) Co-transplantation of Schwann and Bone Marrow Stromal Cells Promotes Locomotor Recovery in the Rat Contusion Model of Spinal Cord Injury. Yakhteh 12: 7.

61. Kamada T, Koda M, Dezawa M, Anahara R, Toyama Y, Yoshinaga K, Hashimoto M, Koshizuka S, Nishio Y, Mannoji C, Okawa A, Yamazaki M (2011) Transplantation of human bone marrow stromal cell-derived Schwann cells reduces cystic cavity and promotes functional recovery after contusion injury of adult rat spinal cord. Neuropathology 31: 48-58.

62. Kamei N, Kwon SM, Alev C, Ishikawa M, Yokoyama A, Nakanishi K, Yamada K, Horii M, Nishimura H, Takaki S, Kawamoto A, Ii M, Akimaru H, Tanaka N, Nishikawa SI, Ochi M, Asahara T (2010) Lnk Deletion Reinforces the Function of Bone Marrow Progenitors in Promoting Neovascularization and Astrogliosis Following Spinal Cord Injury. Stem Cells 28: 365-375.

63. Kang KN, Lee JY, Kim DY, Lee BN, Ahn HH, Lee B, Khang G, Park SR, Min BH, Kim JH, Lee HB, Kim MS (2011) Regeneration of Completely Transected Spinal Cord Using Scaffold of Poly(D,L-Lactide-co-Glycolide)/Small Intestinal Submucosa Seeded with Rat Bone Marrow Stem Cells. Tissue Engineering Part A 17: 2143-2152.

64. Karimi-Abdolrezaee S, Eftekharpour E, Wang J, Morshead CM, Fehlings MG (2006) Delayed transplantation of adult neural precursor cells promotes remyelination and functional neurological recovery after spinal cord injury. J Neurosci 26: 3377-3389.

65. Keirstead HS, Nistor G, Bernal G, Totoiu M, Cloutier F, Sharp K, Steward O (2005) Human embryonic stem cell-derived oligodendrocyte progenitor cell transplants remyelinate and restore locomotion after spinal cord injury. Journal of Neuroscience 25: 4694-4705.

66. Kim DS, Jung SJ, Nam TS, Jeon YH, Lee DR, Lee JS, Leem JW, Kim DW (2010) Transplantation of GABAergic neurons from ESCs attenuates tactile hypersensitivity following spinal cord injury. Stem Cells 28: 2099-2108.

67. Koda M, Okada S, Nakayama T, Koshizuka S, Kamada T, Nishio Y, Someya Y, Yoshinaga K, Okawa A, Moriya H, Yamazaki M (2005) Hematopoietic stem cell and marrow stromal cell for spinal cord injury in mice. Neuroreport 16: 1763-1767.

68. Koshizuka S, Okada S, Okawa A, Koda M, Murasawa M, Hashimoto M, Kamada T, Yoshinaga K, Murakami M, Moriya H, Yamazaki M (2004) Transplanted hematopoietic stem cells from bone marrow differentiate into neural lineage cells and promote functional recovery after spinal cord injury in mice. Journal of Neuropathology & Experimental Neurology 63: 64-72.

69. Lebedev SV, Karasev AV, Chekhonin VP, Savchenko EA, Viktorov IV, Chelyshev YA, Shaimardanova GF (2010) Study of the efficiency of transplantation of human neural stem cells to rats with spinal trauma: the use of functional load tests and BBB test. Bull Exp Biol Med 149: 377-382.

70. Lee KH, Suh-Kim H, Choi JS, Jeun SS, Kim EJ, Kim SS, Yoon DH, Lee BH (2007) Human mesenchymal stem cell transplantation promotes functional recovery following acute spinal cord injury in rats. Acta Neurobiologiae Experimentalis 67: 13-22.

71. Lee KH, Yoon DH, Park YG, Lee BH (2005) Effects of glial transplantation on functional recovery following acute spinal cord injury. Journal of Neurotrauma 22: 575-589.

72. Lee KH, Oh JS, Ha Y, Lee BH, Pennant WA, Yoon DH, Kim KN (2011) Neurotrophin-3-Over-Expressing Mesenchymal Stem Cell Transplantation Improves Functional Recovery and Axonal Outgrowth in a Rat Model of Spinal Cord Injury. Tissue Engineering and Regenerative Medicine 8: 477-481.

73. Li CR, Li W, Cai WQ, Chen DY, Su BY (2004) Effects of neural stem cell transplantation on the functional recovery of rats with spinal cord injury. [Chinese]. Chinese Journal of Clinical Rehabilitation 8: 6364-6366.

74. Li HJ, Liu HY, Zhao ZM, Lu SH, Yang RC, Zhu HF, Cai YL, Zhang QJ, Han ZC (2004) [Transplantation of human umbilical cord stem cells improves neurological function recovery after spinal cord injury in rats]. Zhongguo Yi Xue Ke Xue Yuan Xue Bao 26: 38-42.

75. Li H, Wen Y, Luo Y, Lan X, Wang D, Sun Z, Hu L (2010) Transplantation of bone marrow mesenchymal stem cells into spinal cord injury: a comparison of delivery different times. Zhongguo xiu fu chong jian wai ke za zhi = Zhongguo xiufu chongjian waike zazhi = Chinese journal of reparative and reconstructive surgery 24: 180-184.

76. Li W, Cai WQ, Li CR (2006) Repair of spinal cord injury by neural stem cells modified with BDNF gene in rats. Neuroscience Bulletin 22: 34-40.

77. Li Y, Zhang WM, Wang TH (2011) Optimal Location and Time for Neural Stem Cell Transplantation into Transected Rat Spinal Cord. Cellular and Molecular Neurobiology 31: 407-414.

78. Liang P, Jin LH, Liang T, Liu EZ, Zhao SG (2006) Human neural stem cells promote corticospinal axons regeneration and synapse reformation in injured spinal cord of rats. Chinese Medical Journal 119: 1331-1338.

79. Lin J, Wang C, Wu Z (2010) [Preliminary study on effects of human brain-derived neurotrophic factor gene-modified bone marrow mesenchymal stem cells by intravenous transplantation on structure and function of rat injured spinal cord]. Zhongguo Xiu Fu Chong Jian Wai Ke Za Zhi 24: 982-987.

80. Liu WG, Wang ZY, Huang ZS (2011) Bone marrow-derived mesenchymal stem cells expressing the bFGF transgene promote axon regeneration and functional recovery after spinal cord injury in rats. Neurol Res 33: 686-693.

81. Liu Y, He ZJ, Xu B, Wu QZ, Liu G, Zhu H, Zhong Q, Deng DY, Ai H, Yue Q, Wei Y, Jun S, Zhou G, Gong QY (2011) Evaluation of cell tracking effects for transplanted mesenchymal stem cells with jetPEI/Gd-DTPA complexes in animal models of hemorrhagic spinal cord injury. Brain Res 1391: 24-35.

82. Lu P, Jones LL, Tuszynski MH (2005) BDNF-expressing marrow stromal cells support extensive axonal growth at sites of spinal cord injury. Experimental Neurology 191: 344-360.

83. Macias MY, Syring MB, Pizzi MA, Crowe MJ, Alexanian AR, Kurpad SN (2006) Pain with no gain: Allodynia following neural stem cell transplantation in spinal cord injury. Experimental Neurology 201: 335-348.

84. Marques SA, Almeida FM, Fernandes AM, Souza CdS, Cadilhe DV, Rehen SK, Blanco Martinez AM (2010) Predifferentiated embryonic stem cells promote functional recovery after spinal cord compressive injury. Brain Research 1349: 115-128.

85. Matsuda R, Yoshikawa M, Kimura H, Ouji Y, Nakase H, Nishimura F, Nonaka J, Toriumi H, Yamada S, Nishiofuku M, Moriya K, Ishizaka S, Nakamura M, Sakaki T (2009) Cotransplantation of Mouse Embryonic Stem Cells and Bone Marrow Stromal Cells Following Spinal Cord Injury Suppresses Tumor Development. Cell Transplantation 18: 39-54.

86. McDonald JW, Liu XZ, Qu Y, Liu S, Mickey SK, Turetsky D, Gottlieb DI, Choi DW (1999) Transplanted embryonic stem cells survive, differentiate and promote recovery in injured rat spinal cord. Nature Medicine 5: 1410-1412.

87. McMahon SS, Albermann S, Rooney GE, Shaw G, Garcia Y, Sweeney E, Hynes J, Dockery P, O'Brien T, Windebank AJ, Allsopp TE, Barry FP (2010) Engraftment, migration and differentiation of neural stem cells in the rat spinal cord following contusion injury. Cytotherapy 12: 313-325.

88. Meng XT, Li C, Dong ZY, Liu JM, Li W, Liu Y, Xue H, Chen D (2008) Co-transplantation of bFGF-expressing amniotic epithelial cells and neural stem cells promotes functional recovery in spinal cord-injured rats. Cell Biology International 32: 1546-1558.

89. Mitsui T, Shumsky JS, Lepore AC, Murray M, Fischer I (2005) Transplantation of neuronal and glial restricted precursors into contused spinal cord improves bladder and motor functions, decreases thermal hypersensitivity, and modifies intraspinal circuitry. J Neurosci 25: 9624-9636.

90. Moreno-Manzano V, Rodriguez-Jimenez FJ, Garcia-Rosello M, Lainez S, Erceg S, Calvo MT, Ronaghi M, Lloret M, Planells-Cases R, Sanchez-Puelles JM, Stojkovic M (2009) Activated Spinal Cord Ependymal Stem Cells Rescue Neurological Function. Stem Cells 27: 733-743.

91. Nakamura M, Okano H, Toyama Y, Dai HN, Finn TP, Bregman BS (2005) Transplantation of embryonic spinal cord-derived neurospheres support growth of supraspinal projections and functional recovery after spinal cord injury in the neonatal rat. Journal of Neuroscience Research 81: 457-468.

92. Neuhuber B, Himes BT, Shumsky JS, Gallo G, Fischer I (2005) Axon growth and recovery of function supported by human bone marrow stromal cells in the injured spinal cord exhibit donor variations. Brain Research 1035: 73-85.

93. Neuhuber B, Barshinger AL, Paul C, Shumsky JS, Mitsui T, Fischer I (2008) Stem cell delivery by lumbar puncture as a therapeutic alternative to direct injection into injured spinal cord. Journal of Neurosurgery-Spine 9: 390-399.

94. Nishio Y, Koda M, Kamada T, Someya Y, Yoshinaga K, Okada S, Harada H, Okawa A, Moriya H, Yamazaki M (2006) The use of hemopoietic stem cells derived from human umbilical cord blood to promote restoration of spinal cord tissue and recovery of hindlimb function in adult rats. J Neurosurg Spine 5: 424-433.

95. Nomura H, Zahir T, Kim H, Katayama Y, Kulbatski I, Morshead CM, Shoichet MS, Tator CH (2008) Extramedullary chitosan channels promote survival of transplanted neural stem and progenitor cells and create a tissue bridge after complete spinal cord transection. Tissue Engineering Part A 14: 649-665.

96. Nori S, Okada Y, Yasuda A, Tsuji O, Takahashi Y, Kobayashi Y, Fujiyoshi K, Koike M, Uchiyama Y, Ikeda E, Toyama Y, Yamanaka S, Nakamura M, Okano H (2011) Grafted human-induced pluripotent stem-cell-derived neurospheres promote motor functional recovery after spinal cord injury in mice. Proceedings of the National Academy of Sciences of the United States of America 108: 16825-16830.

97. Ohta M, Suzuki Y, Noda T, Ejiri Y, Dezawa M, Kataoka K, Chou H, Ishikawa N, Matsumoto N, Iwashita Y, Mizuta E, Kuno S, Ide C (2004) Bone marrow stromal cells infused into the cerebrospinal fluid promote functional recovery of the injured rat spinal cord with reduced cavity formation. Exp Neurol 187: 266-278.

98. Olson HE, Rooney GE, Gross L, Nesbitt JJ, Galvin KE, Knight A, Chen B, Yaszemski MJ, Windebank AJ (2009) Neural Stem Cell- and Schwann Cell-Loaded Biodegradable Polymer Scaffolds Support Axonal Regeneration in the Transected Spinal Cord. Tissue Engineering Part A 15: 1797-1805.

99. Osaka M, Honmou O, Murakami T, Nonaka T, Houkin K, Hamada H, Kocsis JD (2010) Intravenous administration of mesenchymal stem cells derived from bone marrow after contusive spinal cord injury improves functional outcome. Brain Research 1343: 226-235.

100. Zlokovic BV, Liu CY (2005) Homologous transplantation of neural stem cells to the injured spinal cord of mice: Comments. Neurosurgery 57: 1024-1025.

101. Pan HC, Cheng FC, Lai SZ, Yang DY, Wang YC, Lee MS (2008) Enhanced regeneration in spinal cord injury by concomitant treatment with granulocyte colony-stimulating factor and neuronal stem cells. Journal of Clinical Neuroscience 15: 656-664.

102. Parr AM, Kulbatski I, Tator CH (2007) Transplantation of adult rat spinal cord stem/progenitor cells for spinal cord injury. Journal of Neurotrauma 24: 835-845.

103. Parr AM, Kulbatski I, Zahir T, Wang X, Yue C, Keating A, Tator CH (2008) Transplanted adult spinal cord-derived neural stem/progenitor cells promote early functional recovery after rat spinal cord injury. Neuroscience 155: 760-770.

104. Parr AM, Kulbatski I, Wang XH, Keating A, Tator CH (2008) Fate of transplanted adult neural stem/progenitor cells and bone marrow-derived mesenchymal stromal cells in the injured adult rat spinal cord and impact on functional recovery. Surgical Neurology 70: 600-607.

105. Pedram MS, Dehghan MM, Soleimani M, Sharifi D, Marjanmehr SH, Nasiri Z (2010) Transplantation of a combination of autologous neural differentiated and undifferentiated mesenchymal stem cells into injured spinal cord of rats. Spinal Cord 48: 457-463.

106. Pu Y, Guo QS, Wang AM, Wu SY, Xing SX, Zhang ZR (2007) Repair of acutely injured spinal cord through constructing tissue-engineered neural complex in adult rats. SO - Chinese Journal of Traumatology - English Edition. 10(3)(pp 171-176), 2007. Date of Publication: 01 Jun 2007.

107. Pu Y, Guo Q-S, Wang A-M, Wu S-Y, Xing S-X, Zhang Z-R (2007) Repair of acutely injured spinal cord through constructing tissue-engineered neural complex in adult rats. Chinese Journal of Traumatology - English Edition 10: 171-176.

108. Qin M-L, Yang W-B, Li H-L, Li C-R, Liu J-J (2008) Effects of neurotrophin-3 modified embryonic stem cells transplantation on the recovery of spinal cord injury. [Chinese]. Journal of Clinical Rehabilitative Tissue Engineering Research 12: 2263-2266.

109. Rossi SL, Nistor G, Wyatt T, Yin HZ, Poole AJ, Weiss JH, Gardener MJ, Dijkstra S, Fischer DF, Keirstead HS (2010) Histological and Functional Benefit Following Transplantation of Motor Neuron Progenitors to the Injured Rat Spinal Cord. PLoS One 5.

110. Saporta S, Kim JJ, Willing AE, Fu ES, Davis CD, Sanberg PR (2003) Human umbilical cord blood stem cells infusion in spinal cord injury: engraftment and beneficial influence on behavior. J Hematother Stem Cell Res 12: 271-278.

111. Setoguchi T, Nakashima K, Takizawa T, Yanagisawa M, Ochiai W, Okabe M, Yone K, Komiya S, Taga T (2004) Treatment of spinal cord injury by transplantation of fetal neural precursor cells engineered to express BMP inhibitor. Exp Neurol 189: 33-44.

112. Shang AJ, Hong SQ, Xu Q, Wang HY, Yang Y, Wang ZF, Xu BN, Jiang XD, Xu RX (2011) NT-3-secreting human umbilical cord mesenchymal stromal cell transplantation for the treatment of acute spinal cord injury in rats. Brain Research 1391: 102-113.

113. Shen J, Zhong XM, Duan XH, Cheng LN, Hong GB, Bi XB, Liu Y (2009) Magnetic Resonance Imaging of Mesenchymal Stem Cells Labeled with Dual (MR and Fluorescence) Agents in Rat Spinal Cord Injury. Academic Radiology 16: 1142-1154.

114. Sheth RN, Manzano G, Li XM, Levi AD (2008) Transplantation of human bone marrow-derived stromal cells into the contused spinal cord of nude rats - Laboratory investigation. Journal of Neurosurgery-Spine 8: 153-162.

115. Someya Y, Koda M, Dezawa M, Kadota T, Hashimoto M, Kamada T, Nishio Y, Kadota R, Mannoji C, Miyashita T, Okawa A, Yoshinaga K, Yamazaki M (2008) Reduction of cystic cavity, promotion of axonal regeneration and sparing, and functional recovery with transplanted bone marrow stromal cell-derived Schwann cells after contusion injury to the adult rat spinal cord. Journal of Neurosurgery-Spine 9: 600-610.

116. Sun Y, Shi J, Fu S, Lu Pei-hua (neuron@shsmu ec, Xu X (2003) Effects of embryonic neural stem cells and glial cell line-derived neurotrophic factor in the repair of spinal cord injury. Shengli Xuebao 55: 349-354.

117. Takenaga M, Ohta Y, Tokura Y, Hamaguchi A, Suzuki N, Nakamura M, Okano H, Igarashi R (2007) Plasma as a scaffold for regeneration of neural precursor cells after transplantation into rats with spinal cord injury. Cell Transplant 16: 57-65.

118. Takeuchi H, Natsume A, Wakabayashi T, Aoshima C, Shimato S, Ito M, Ishii J, Maeda Y, Hara M, Kim SU, Yoshida J (2007) Intravenously transplanted human neural stem cells migrate to the injured spinal cord in adult mice in an SDF-1- and HGF-dependent manner. Neuroscience Letters 426: 69-74.

119. Tarasenko YI, Gao J, Nie L, Johnson KM, Grady JJ, Hulsebosch CE, McAdoo DJ, Wu P (2007) Human fetal neural stem cells grafted into contusion-injured rat spinal cords improve behavior. Journal of Neuroscience Research 85: 47-57.

120. Teng YD, Lavik EB, Qu XL, Park KI, Ourednik J, Zurakowski D, Langer R, Snyder EY (2002) Functional recovery following traumatic spinal cord injury mediated by a unique polymer scaffold seeded with neural stem cells. Proceedings of the National Academy of Sciences of the United States of America 99: 3024-3029.

121. Urdzikova L, Jendelova P, Glogarova K, Burian M, Hajek M, Sykova E (2006) Transplantation of bone marrow stem cells as well as mobilization by granulocyte-colony stimulating factor promotes recovery after spinal cord injury in rats. Journal of Neurotrauma 23: 1379-1391.

122. Wang C, Lin J, Wu C, Chen R (2011) Adenovirus-mediated human brain-derived neurotrophic factor gene-modified bone marrow mesenchymal stem cell transplantation for spinal cord injury. Neural Regeneration Research 6: 1211-1216.

123. Wang D, Wen Y, Lan X, Li H (2010) Experimental study on bone marrow mesenchymal stem cells seeded in chitosan-alginate scaffolds for repairing spinal cord injury. Zhongguo xiu fu chong jian wai ke za zhi = Zhongguo xiufu chongjian waike zazhi = Chinese journal of reparative and reconstructive surgery 24: 190-196.

124. Wang D, Yang Z, Zhang J (2010) Treatment of spinal cord injury by mild hypothermia combined with bone marrow mesenchymal stem cells transplantation in rats. Zhongguo xiu fu chong jian wai ke za zhi = Zhongguo xiufu chongjian waike zazhi = Chinese journal of reparative and reconstructive surgery 24: 801-805.

125. Wang D, Kang D, Lin J, Yu L, Lin Z, Wu Z (2010) Combination of bone marrow mesenchymal stem cells and brain-derived neurotrophic factor for treating spinal cord injury. Neural Regeneration Research 5: 491-495.

126. Wang G, Ao Q, Gong K, Zuo H, Gong Y, Zhang X (2010) Synergistic Effect of Neural Stem Cells and Olfactory Ensheathing Cells on Repair of Adult Rat Spinal Cord Injury. Cell Transplantation 19: 1325-1337.

127. Wang T, Li L-S, Liu Y-P, Qu J-F, Xu H-W (2007) Transplanted time of dermal multipotent stem cells following spinal cord injury. [Chinese]. Journal of Clinical Rehabilitative Tissue Engineering Research 11: 6615-6619.

128. Wang T, Ren X, Xiong J, Zhang L, Qu J, Xu W (2011) Tailless-like (TLX) protein promotes neuronal differentiation of dermal multipotent stem cells and benefits spinal cord injury in rats. Cell Mol Neurobiol 31: 479-487.

129. Watanabe K, Nakamura M, Iwanami A, Fujita Y, Kanemura Y, Toyama Y, Okano H (2004) Comparison between fetal spinal-cord- and forebrain-derived neural stem/progenitor cells as a source of transplantation for spinal cord injury. Developmental Neuroscience 26: 275-287.

130. Wu SF, Suzuki Y, Ejiri Y, Noda T, Bai HL, Kitada M, Kataoka K, Ohta M, Chou H, Ide C (2003) Bone marrow stromal cells enhance differentiation of cocultured neurosphere cells and promote regeneration of injured spinal. Journal of Neuroscience Research 72: 343-351.

131. Wu W, Zhao H, Xie B, Liu H, Chen Y, Jiao G, Wang H (2011) Implanted spike wave electric stimulation promotes survival of the bone marrow mesenchymal stem cells and functional recovery in the spinal cord injured rats. Neuroscience Letters 491: 73-78.

132. Wu ZY, Hui GZ, Lu Y, Wu X, Guo LH (2006) Transplantation of human amniotic epithelial cells improves hindlimb function in rats with spinal cord injury. Chinese Medical Journal 119: 2101-2107.

133. Xu G, Li X, Bai J, Cai Q, Li L, Shen L (2003) [Functional evaluation for neural progenitor cells transplantation to treat spinal cord injury in rats]. Beijing Da Xue Xue Bao 35: 274-276.

134. Xu G, Li X, Bai Y, Bai J, Li L, Shen L (2004) Improving recovery of spinal cord-injured rats by telomerase-driven human neural progenitor cells. Restor Neurol Neurosci 22: 469-476.

135. Xu CJ, Xu L, Huang LD, Li Y, Yu PP, Hang Q, Xu XM, Lu PH (2011) Combined NgR vaccination and neural stem cell transplantation promote functional recovery after spinal cord injury in adult rats. Neuropathol Appl Neurobiol 37: 135-155.

136. Yan Q, Ruan Jw, Ding Y, Li Wj, Li Y, Zeng Ys (2011) Electro-acupuncture promotes differentiation of mesenchymal stem cells, regeneration of nerve fibers and partial functional recovery after spinal cord injury. Experimental and Toxicologic Pathology 63: 151-156.

137. Yang CC, Shih YH, Ko MH, Hsu SY, Cheng H, Fu YS (2008) Transplantation of human umbilical mesenchymal stem cells from Wharton's jelly after complete transection of the rat spinal cord. PLoS One 3: e3336.

138. Yan JH, Wang FH, Li CD, Wang Y, Ma J (2007) Transplantation of neural precursors derived from embryonic stem cells for repairing spinal cord injury. SO - Journal of Clinical Rehabilitative Tissue Engineering Research. 11(24)(pp 4842-4846), 2007. Date of Publication: 17 Jun 2007.

139. Yano S, Kuroda S, Shichinohe H, Seki T, Ohnishi T, Tamagami H, Hida K, Iwasaki Y (2006) Bone marrow stromal cell transplantation preserves gammaaminobutyric acid receptor function in the injured spinal cord. J Neurotrauma 23: 1682-1692.

140. Yin G, Tang X, Lin Y (2006) Recovery of adult rat spinal cord injury by co-transplantion of human embryonic olfactory ensheathing cells and neuro stem cells. [Chinese] SO - Chinese Journal of Rehabilitation Medicine. 21(8)(pp 680-682+719), 2006. Date of Publication: Aug 2006.

141. Yoshihara H, Shumsky JS, Neuhuber B, Otsuka T, Fischer I, Murray M (2006) Combining motor training with transplantation of rat bone marrow stromal cells does not improve repair or recovery in rats with thoracic contusion injuries. Brain Research 1119: 65-75.

142. Yoshihara T, Ohta M, Itokazu Y, Matsumoto N, Dezawa M, Suzuki Y, Taguchi A, Watanabe Y, Adachi Y, Ikehara S, Sugimoto H, Ide C (2007) Neuroprotective effect of bone marrow-derived mononuclear cells promoting functional recovery from spinal cord injury. Journal of Neurotrauma 24: 1026-1036.

143. Yu D, Lu G, Cao Y, Li G, Zhi X, Fan Z (2011) Effects of bone marrow mesenchymal stem cells transplantation on expression of vascular endothelial growth factor gene and angiogenesis after spinal cord injury in rats. Zhongguo xiu fu chong jian wai ke za zhi = Zhongguo xiufu chongjian waike zazhi = Chinese journal of reparative and reconstructive surgery 25: 837-841.

144. Zhang HT, Yang HL, Qu J, Zhang J, Meng B, Zhang ZG, Tang TS, Xu YZ, Jiang WM (2007) Vein transplantation of human umbilical cord blood mesenchymal stem cells improves neurological function recovery after spinal cord injury in rats. [Chinese] SO - Journal of Clinical Rehabilitative Tissue Engineering Research. 11(15)(pp 2886-2889), 2007. Date of Publication: 15 Apr 2007.

145. Zhang HT, Cheng HY, Cai YQ, Ma X, Liu WP, Yan ZJ, Jiang XD, Xu RX (2009) Comparison of adult neurospheres derived from different origins for treatment of rat spinal cord injury. Neuroscience Letters 458: 116-121.

146. Zhang H, Yang H, Zhang H, Qu J (2010) Functional recovery and microenvironmental alterations in a rat model of spinal cord injury following human umbilical cord blood-derived mesenchymal stem cells transplantation. Neural Regeneration Research 5: 165-170.

147. Zhang J, Wu G, Zhao F, Jin X (2010) Co-transplantation of Schwann cells and bone marrow stromal cells versus single cell transplantation on repairing hemisected spinal cord injury of rats. Neural Regeneration Research 5: 805-813.

148. Zhang LY, Gu ST, Zhao CP, Wen TQ (2007) Combined treatment of neurotrophin-3 gene and neural stem cells is propitious to functional recovery after spinal cord injury. Cell Transplantation 16: 475-481.

149. Zhang W, Yan Q, Zeng YS, Zhang XB, Xiong Y, Wang JM, Chen SJ, Li Y, Bruce IC, Wu W (2010) Implantation of adult bone marrow-derived mesenchymal stem cells transfected with the neurotrophin-3 gene and pretreated with retinoic acid in completely transected spinal cord. Brain Res 1359: 256-271.

150. Zhang XB, Zeng YS, Zhang W, Wang JM, Wu JL, Li J (2007) Co-transplantation of neural stem cells and NT-3-overexpressing Schwann cells in transected spinal cord. Journal of Neurotrauma 24: 1863-1877.

151. Zhang Z, Wen Y, Zhang ZS, Wen YM (2010) Bone marrow mesenchymal stem cells derived neuron-like cells for spinal cord injury of adult rats. Progress in Modern Biomedicine 10: 4227-4263.

152. Zhao ZM, Li HJ, Liu HY, Lu SH, Yang RC, Zhang QJ, Han ZC (2004) Intraspinal transplantation of CD34+ human umbilical cord blood cells after spinal cord hemisection injury improves functional recovery in adult rats. Cell Transplant 13: 113-122.

153. Zhao P, Feng S, Wang Y, Zhao P, Feng SQ, Wang Y (2010) Effect of different concentration of human umbilical cord mesenchymal stem cells in experimental spinal cord injury in rats. Zhongguo Jiaoxing Waike Zazhi / Orthopedic Journal of China 18: 1817-1825.

154. Yu Q, Bai Ys, Lin J (2010) Effect of astragalus injection combined with mesenchymal stem cells transplantation for repairing the Spinal cord injury in rats. Zhongguo Zhong xi yi jie he za zhi Zhongguo Zhongxiyi jiehe zazhi = Chinese journal of integrated traditional and Western medicine / Zhongguo Zhong xi yi jie he xue hui, Zhongguo Zhong yi yan jiu yuan zhu ban 30: 393-397.

155. Zhilai Z, Hui Z, Yinhai C, Zhong C, Shaoxiong M, Bo Y, Anmin J (2011) Combination of NEP 1-40 infusion and bone marrow-derived neurospheres transplantation inhibit glial scar formation and promote functional recovery after rat spinal cord injury. Neurol India 59: 579-585.

156. Ziv Y, Avidan H, Pluchino S, Martino G, Schwartz M (2006) Synergy between immune cells and adult neural stem/progenitor cells promotes functional recovery from spinal cord injury (vol 103, pg 13174, 2006). Proceedings of the National Academy of Sciences of the United States of America 103.
